# Comparison of tumor‐informed and tumor‐naïve sequencing assays for ctDNA detection in breast cancer

**DOI:** 10.15252/emmm.202216505

**Published:** 2023-05-10

**Authors:** Angela Santonja, Wendy N Cooper, Matthew D Eldridge, Paul A W Edwards, James A Morris, Abigail R Edwards, Hui Zhao, Katrin Heider, Dominique‐Laurent Couturier, Aadhitthya Vijayaraghavan, Paulius Mennea, Emma‐Jane Ditter, Christopher G Smith, Chris Boursnell, Raquel Manzano García, Oscar M Rueda, Emma Beddowes, Heather Biggs, Stephen‐John Sammut, Nitzan Rosenfeld, Carlos Caldas, Jean E Abraham, Davina Gale

**Affiliations:** ^1^ Cancer Research UK Cambridge Institute, University of Cambridge, Li Ka Shing Centre Cambridge UK; ^2^ Cancer Research UK Cambridge Centre, Cancer Research UK Cambridge Institute, Li Ka Shing Centre Cambridge UK; ^3^ Department of Pathology University of Cambridge Cambridge UK; ^4^ MRC Biostatistics Unit University of Cambridge Cambridge UK; ^5^ Department of Oncology University of Cambridge Cambridge UK; ^6^ Precision Breast Cancer Institute, Cambridge University Hospitals NHS Foundation Trust, Addenbrooke's Hospital Cambridge UK

**Keywords:** circulating tumor DNA, liquid biopsy, hybrid capture, multiplex PCR, whole‐genome sequencing, Cancer, Chromatin, Transcription & Genomics

## Abstract

Analysis of circulating tumor DNA (ctDNA) to monitor cancer dynamics and detect minimal residual disease has been an area of increasing interest. Multiple methods have been proposed but few studies have compared the performance of different approaches. Here, we compare detection of ctDNA in serial plasma samples from patients with breast cancer using different tumor‐informed and tumor‐naïve assays designed to detect structural variants (SVs), single nucleotide variants (SNVs), and/or somatic copy‐number aberrations, by multiplex PCR, hybrid capture, and different depths of whole‐genome sequencing. Our results demonstrate that the ctDNA dynamics and allele fractions (AFs) were highly concordant when analyzing the same patient samples using different assays. Tumor‐informed assays showed the highest sensitivity for detection of ctDNA at low concentrations. Hybrid capture sequencing targeting between 1,347 and 7,491 tumor‐identified mutations at high depth was the most sensitive assay, detecting ctDNA down to an AF of 0.00024% (2.4 parts per million, ppm). Multiplex PCR targeting 21–47 tumor‐identified SVs per patient detected ctDNA down to 0.00047% AF (4.7 ppm) and has potential as a clinical assay.

## Graphical abstract

Tumor‐informed and tumor‐naïve assays were developed to compare detection of circulating tumor DNA in serial plasma samples from patients with stage I–IV breast cancer. These assays targeted structural variants (SVs), single nucleotide variants (SNVs), and somatic copy‐number aberrations (SCNAs).

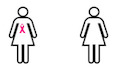

**The paper explained**

**Problem**

Circulating tumor DNA (ctDNA) can be used as a non‐invasive liquid biopsy in cancer patients to track disease burden in blood. Different strategies have been used to quantify ctDNA, but few studies have compared the performance of different tumor‐informed and tumor‐naïve assays to detect ctDNA in the same patient samples.

**Results**

Our results demonstrate that ctDNA dynamics and tumor allele fractions were highly concordant when targeting different mutation types in serial blood samples collected from breast cancer patients undergoing treatment. Tumor‐informed assays showed the highest sensitivity for detection of ctDNA at low concentrations. SNV‐hybrid capture, targeting thousands of single nucleotide variants, and SV‐multiplex PCR, targeting tens of structural variants, were able to detect ctDNA down to a few parts per million.

**Impact**

Choice of assay for ctDNA quantification depends on many factors including the required sensitivity for its intended use, the mutation type being assayed, turnaround time, and cost. This study demonstrates that personalized assays targeting patient‐specific mutations identified in the tumor were the most sensitive assays to detect low levels of ctDNA in blood, and SV‐multiplex PCR has potential to be used as a clinical diagnostic assay.

## Introduction

Circulating tumor DNA (ctDNA) has emerged as an effective and minimally invasive biomarker for molecular profiling and the stratification of patients to targeted therapy, detection of minimal residual disease (MRD), and monitoring tumor growth and treatment response (Wan *et al*, 2017; Corcoran & Chabner, 2018). ctDNA is comprised of fragments of tumor‐derived DNA that can be found in the plasma of cancer patients and often represents a small fraction of the total circulating cell‐free DNA. ctDNA levels can vary in different cancer types, and higher fractional concentrations of ctDNA in plasma have been associated with larger tumor volume and more advanced disease stages (Bettegowda *et al*, 2014; Newman *et al*, 2014; Parkinson *et al*, 2016). In patients with early‐stage cancer or with residual disease following treatment, ctDNA fractions can be very low (< 0.01% variant allele fraction) and its detection can be challenging, requiring sensitive methods for analysis (Gale *et al*, 2022).

In recent years, different methods have been developed to detect ctDNA and to distinguish it from non‐cancerous cell‐free DNA derived from other cells by analysis of tumor‐specific genomic alterations including single nucleotide variants (SNVs), structural variants (SVs), and somatic copy‐number aberrations (SCNAs). Techniques include digital PCR, targeted next‐generation sequencing (NGS) using multiplex PCR‐based amplification or hybrid capture, and whole‐genome sequencing (WGS) (Diehl *et al*, 2008; Leary *et al*, 2010; Forshew *et al*, 2012; Heitzer *et al*, 2013; Garcia‐Murillas *et al*, 2015; Newman *et al*, 2016). Additional studies have used epigenetic analysis to profile differentially methylated regions in cell‐free DNA and identify the tissue of origin (Shen *et al*, 2019; Liu *et al*, 2020; Sadeh *et al*, 2021). In patients with low‐burden disease, the detection of ctDNA when only a few tumor‐derived molecules may be present in blood can be challenging and is often limited by stochastic sampling (Wan *et al*, 2017). In recent years, the use of next‐generation sequencing and advanced error‐suppression methods, including the incorporation of unique molecular identifiers (UMIs) to tag individual molecules, have enabled assays to become increasingly more sensitive. Sensitivity can be improved further by analysis of a higher number of ctDNA molecules either through the use of larger volumes of blood, minimizing the loss of molecules during library preparation steps, and/or targeting a higher number of tumor‐specific mutations (Newman *et al*, 2016; Vollbrecht *et al*, 2018; McDonald *et al*, 2019; Streubel *et al*, 2019; Wan *et al*, 2020, 2021; Zviran *et al*, 2020; Kurtz *et al*, 2021; Flach *et al*, 2022; Gale *et al*, 2022).

Using a tumor‐informed approach, by prior sequencing of tumor biopsy samples to identify multiple patient‐specific mutations, the development of personalized assays has enabled sensitive detection of SNVs (Coombes *et al*, 2019; McDonald *et al*, 2019; Wan *et al*, 2020; Magbanua *et al*, 2021). Wan et al. reliably detected ctDNA at an allele fraction (AF) of 0.001% using such an approach, coupled with analysis involving the INtegration of VAriant Reads (INVAR), which combines error‐suppression and signal‐enrichment methods based on the fragment size of ctDNA to enhance the sensitivity of detection (Wan *et al*, 2020). In order to increase the number of loci analyzed, studies have interrogated somatic copy‐number aberrations (SCNAs) using shallow whole‐genome sequencing (sWGS), a tumor‐naïve approach that does not require prior tumor‐sequencing information (Heitzer *et al*, 2013; Douville *et al*, 2020). Structural variants (SVs) have been less well studied but have the potential to be highly sensitive and specific biomarkers. SVs present in tumor DNA consist of rearrangements of genomic sequences that do not naturally occur in normal cells; therefore, their analysis can avoid confounding background signal which is observed when analyzing single‐base changes that may result from PCR and/or sequencing errors. However, identifying SVs remains a challenge and there are much fewer events in the genome compared to SNVs (Li *et al*, 2020). Clinical assays have been developed that target disease‐specific structural rearrangements such as *ALK* and *ROS1* gene fusions, which have enabled non‐small‐cell lung cancer (NSCLC) patients harboring these mutations to be effectively treated with tyrosine kinase inhibitors (Lanman *et al*, 2015; Plagnol *et al*, 2018). Two early studies demonstrated the ability to analyze ctDNA using patient‐specific SVs (Leary *et al*, 2010; McBride *et al*, 2010), however, this approach has not currently been widely adopted. Once identified by prior tumor sequencing, assays targeting SVs can be applied for monitoring ctDNA (Olsson *et al*, 2015; Harris *et al*, 2016; Kim *et al*, 2019).

While a wide range of techniques can be used for ctDNA analysis, there have been few studies comparing the sensitivity of detection in the same samples to assess their relative performance (Thress *et al*, 2015; Kuderer *et al*, 2017; Stetson *et al*, 2019). Here, we have developed different assays to detect and quantify ctDNA in plasma samples from patients with early‐ and late‐stage breast cancer, targeting a range of genomic alterations (SVs, SNVs, and SCNAs) and using different methods for library preparation. We applied tumor‐informed targeted sequencing approaches to analyze patient‐specific SVs and/or SNVs using multiplex PCR (SV‐multiplex PCR) and hybrid capture (SV‐hybrid capture and SNV‐hybrid capture). In addition, we used whole‐genome sequencing at different sequencing depths of coverage (sWGS: shallow WGS at 1.2× mean coverage; modWGS: moderately deep WGS at 20× mean coverage; and deepWGS, at 399× mean coverage) to analyze patient‐specific SVs (SV‐modWGS and SV‐deepWGS) and SNVs (SNV‐modWGS and SNV‐deepWGS), as well as a tumor‐naïve approach to analyze copy‐number aberrations (SCNA‐sWGS, SCNA‐modWGS, and SCNA‐deepWGS).

## Results

### Patients and samples

Primary or metastatic tumor tissue (*n* = 7), matched buffy coat (*n* = 7), and serial plasma samples (*n* = 54) were collected from seven patients with breast cancer, with either early‐stage (*n* = 4) or late‐stage (*n* = 3) disease, recruited to the Personalized Breast Cancer Programme (PBCP) (Table EV1). As controls, we used 19 plasma samples from healthy donors (18 samples from individual donors and one pool from five individuals).

**Figure 1:**
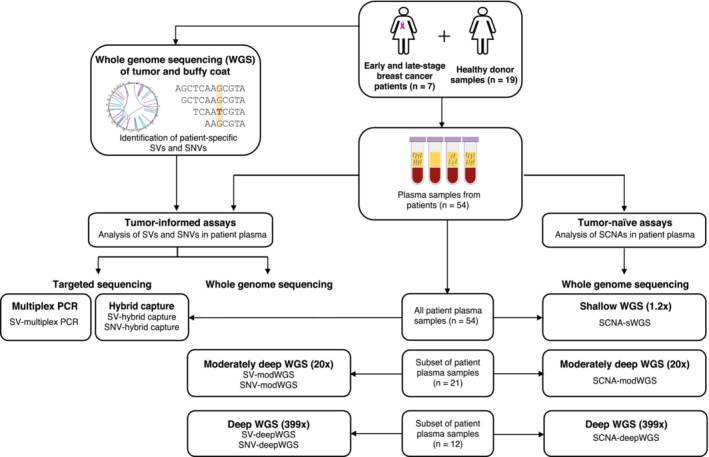
Overview of the study design Longitudinal plasma samples (*n* = 54) from seven breast cancer patients, with either early‐stage (*n* = 4) or late‐stage (*n* = 3) disease, were analyzed using different ctDNA assays. As controls, plasma samples (*n* = 19) from healthy donors were analyzed. Whole‐genome sequencing (WGS) of tumor and matched buffy coat DNA was first performed to identify patient‐specific structural variants (SVs) and single nucleotide variants (SNVs). These were used to define tumor‐informed ctDNA assays including targeted sequencing and whole‐genome sequencing (WGS) to different depths of sequencing. Targeted sequencing to evaluate SVs and SNVs (SV‐multiplex PCR, SV‐hybrid capture, and SNV‐hybrid capture) was performed in all 54 patient plasma samples. WGS at various depths was performed in a subset of 21 samples that were then downsampled to 600 M reads (modWGS, mean coverage depth 20×) prior to further analyses; these included 12 samples with deep sequencing (deepWGS, mean coverage depth 399×) analyzed before and after downsampling. As a tumor‐informed approach, SVs and SNVs were analyzed in the modWGS (SV‐modWGS and SNV‐modWGS) and the deepWGS assays (SV‐deepWGS and SNV‐deepWGS). Somatic copy‐number aberrations (SCNAs) were evaluated using WGS as a tumor‐naïve approach (not requiring prior knowledge of the tumor) by shallow WGS (SCNA‐sWGS, mean coverage depth 1.2×, 54 samples), modWGS (SCNA‐modWGS, 21 samples), and deepWGS (SCNA‐deepWGS, 12 samples).

The study design is shown in Fig 1. Whole‐genome sequencing (WGS) was performed on DNA extracted from either primary or metastatic tumor tissue and on germline DNA from each patient. Patient‐specific SVs and SNVs were identified and used to design tumor‐informed assays to detect ctDNA in plasma samples, either by targeted sequencing (multiplex PCR and hybrid capture) or by WGS at different depths of coverage (sWGS, modWGS, and deepWGS). Tumor‐informed approaches included the analysis of SVs by multiplex PCR followed by sequencing (SV‐multiplex PCR), and the analysis of both SVs and SNVs in whole‐genome sequencing data at different depths (SV‐modWGS, SNV‐modWGS, SV‐deepWGS, and SNV‐deepWGS) and from targeted regions captured by hybridization (SV‐hybrid capture and SNV‐hybrid capture). Multiplex PCR, hybrid capture, and sWGS were used to analyze a total of 54 plasma samples collected from the seven patients during the course of treatment. ModWGS was performed on a subset of 21 samples that were downsampled to 600 M reads, which included 12 samples that were also analyzed by deepWGS (mean coverage depth 399×) prior to downsampling. SCNAs were evaluated using a tumor‐naïve approach (SCNA‐sWGS, SCNA‐modWGS, and SCNA‐deepWGS).

### Whole‐genome sequencing of tumor and germline buffy coat DNA to identify patient‐specific SVs and SNVs

Whole‐genome PCR‐free libraries were prepared from tumor and matched buffy coat DNA from the seven breast cancer patients, and sequenced on a HiSeqX (Illumina). The median unique sequencing coverage depth was 116× for tumor and 39× for buffy coat samples (Table EV2).

**Figure EV1:**
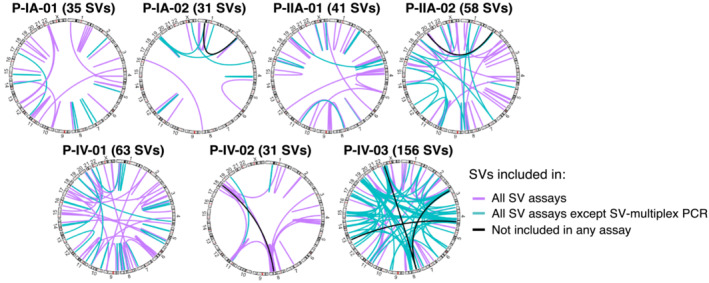
Circos plots showing patient‐specific SVs (structural variants) from all breast cancer patients Plots show the number and location of all patient‐specific SVs identified by tumor WGS (number in brackets), which were subsequently targeted by the different ctDNA assays (see Table EV3). The breakpoints of all SVs depicted (except those indicated in black) were confirmed by multiplex PCR or hybrid capture. Purple lines indicate SVs included in all SV assays (SV‐multiplex PCR, SV‐hybrid capture, SV‐modWGS, and SV‐deep‐WGS). Turquoise lines indicate SVs analyzed only by SV‐hybrid capture, SV‐modWGS, and SV‐deepWGS but not by SV‐multiplex PCR. Black lines indicate six SVs identified by WGS of the tumor tissue that was not detected in plasma using any assay (due to no reads observed or homology with repetitive regions) and therefore was removed from further analysis.

Structural variants were identified by analysis of WGS tumor and buffy coat data using Manta (Chen *et al*, 2016). A total of 415 high‐confidence SVs (31–156 per patient) were identified by matching calls to copy‐number steps (breakpoints or transitions). The predicted SV sequences were then evaluated by the analysis of sequencing data from tumor tissue and matched buffy coats from all patients by SV‐multiplex PCR and SV‐hybrid capture. The precise breakpoint sequences were confirmed in 99% (409/415) of the SVs. The remaining six SVs included four with no reads observed in tumor tissue or any plasma sample using any of the assays (P‐IA‐02_R209, P‐IV‐02_R019, P‐IV‐03_R253, and P‐IV‐03_R295), and two that had non‐specific amplification, with high homology to Alu/SINE repeat regions (P‐IIA‐02_R048 and P‐IV‐03_R353); P‐IIA‐02_R048 was the only SV with sequencing reads observed in buffy‐coat DNA. These six SVs were excluded from further analyses in any assay (Tables EV2 and EV3). The Circos plots (Gu *et al*, 2014) shown in Fig EV1 detail the patient‐specific SVs identified in each of the seven patients and indicate which were targeted by the different ctDNA assays.

**Figure 2:**
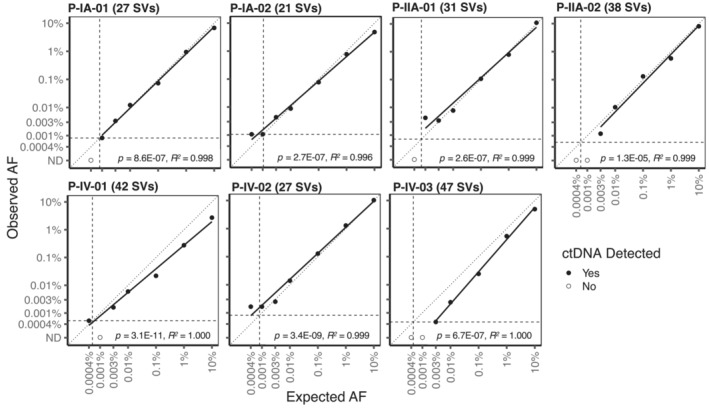
Relationship between observed and expected allele fractions (AFs) in tumor dilution series with SV‐multiplex PCR Numbers in brackets indicate the number of SVs (structural variants) targeted by SV‐multiplex PCR in each patient. Tumor dilutions ranged from 0.0004% to 10% AF. The vertical and horizontal dashed lines represent the theoretical limit of detection of each assay based on the number of analyzed SVs and input cell‐free DNA copies. The diagonal dotted line represents the unit line. The solid black line shows the linear regression fit. The *P*‐values of the slope parameter Wald *t*‐tests as well as the linear model‐fit *R*‐squared estimates are also indicated.

The WGS tumor data were analyzed in comparison to the matched buffy coat using Mutect2 (Broad Institute, 2022) and Strelka (Saunders *et al*, 2012) variant callers. Low‐complexity SNVs were excluded as well as variants present within the gnomAD germline resource (Karczewski *et al*, 2020) or in a pool of buffy coat samples from 200 breast cancer patients. After applying the selected filters (see Methods), between 4,153 and 16,015 SNVs were identified per patient (Tables EV2 and EV4).

### Tumor‐informed assays: ctDNA assays targeting SVs and SNVs

#### SV‐multiplex PCR assays

##### SV‐multiplex PCR: Assay optimization and performance testing using a tumor dilution series

Patient‐specific SV‐multiplex PCR assays were developed for each patient using primers designed to flank the SV breakpoint sequences and were tested on tumor and buffy coat. One primer pair (P‐IIA‐02_R048) was removed following testing in single plex due to amplification observed when using buffy coat DNA as template. As a result of testing in multiplex, 53 primer pairs were removed due to either no SV reads generated in amplification of tumor DNA from the appropriate patient or due to off‐target reads observed. The optimized patient‐specific primer pairs were included in the final SV‐multiplex assay pools, each targeting between 21 and 47 patient‐specific SVs (Fig EV1, Tables EV2 and EV5) and *RPP30_97bp* (amplifying a 97 bp region from the *RPP30* housekeeper gene) as an internal positive control.

To assess the performance of the seven patient‐specific SV‐multiplex PCR assays, a tumor DNA dilution series experiment was performed for each patient‐specific assay, testing a range of tumor DNA dilutions between 10% AF and 0.0004% AF and using matched buffy coat and no template control (NTC; water) as negative controls. Then, patient‐specific SV‐multiplex PCR assays were performed on the 54 patient plasma samples, using plasma from healthy donors (a pool of five individuals) and NTC as negative controls. The number of samples assayed and paired reads generated per library are shown in Table EV6.

Data generated by analysis of the tumor dilution series are shown in Table EV7, including the observed and expected AFs for each dilution and number of SVs detected in each well in which every dilution was divided. ctDNA was not detected in buffy coats or NTCs. The correlation between the observed and expected AFs of each sample using the different patient‐specific assays is shown in Fig 2. Linear regression analysis indicated that all the patient‐specific SV‐multiplex PCR assays are quantitative and linearly related (Fig 2). The theoretical limit of detection for each of the patient‐specific SV‐multiplex PCR assays is linearly related to the number of patient‐specific SVs targeted and the number of input cell‐free DNA amplifiable copies and was calculated as [1/(4,500 input cell‐free DNA copies × number of patient‐specific SVs targeted)]. The theoretical limit of detection ranges from 0.00047% AF (P‐IV‐03, 47 SVs targeted) to 0.0011% AF (P‐IA‐02, 21 SVs targeted), and is represented with the horizontal and vertical dotted lines in Fig 2. In all patients, ctDNA was detected in tumor dilutions with an expected AF down to 0.003%. Below this allele fraction, the number of patient‐specific SVs expected to be detected ranged between 0 and 2, therefore, the detection of tumor DNA and the AF quantification in these dilutions are subject to sampling error. In tumor dilutions at an expected AF of 0.001%, ctDNA was detected in four of seven patients (observed AF of 0.0008% in P‐IA‐01, 0.001% in P‐IA‐02, 0.004% in P‐IIA‐01, and 0.002% in P‐IV‐02). In tumor dilutions at an expected AF of 0.0004%, ctDNA was detected in three of seven patients (observed AF of 0.001% in P‐IA‐02, 0.0005% in P‐IV‐01, and 0.002% in P‐IV‐02).

##### Analysis of serial plasma samples using the SV‐multiplex PCR assays

**Figure 3:**
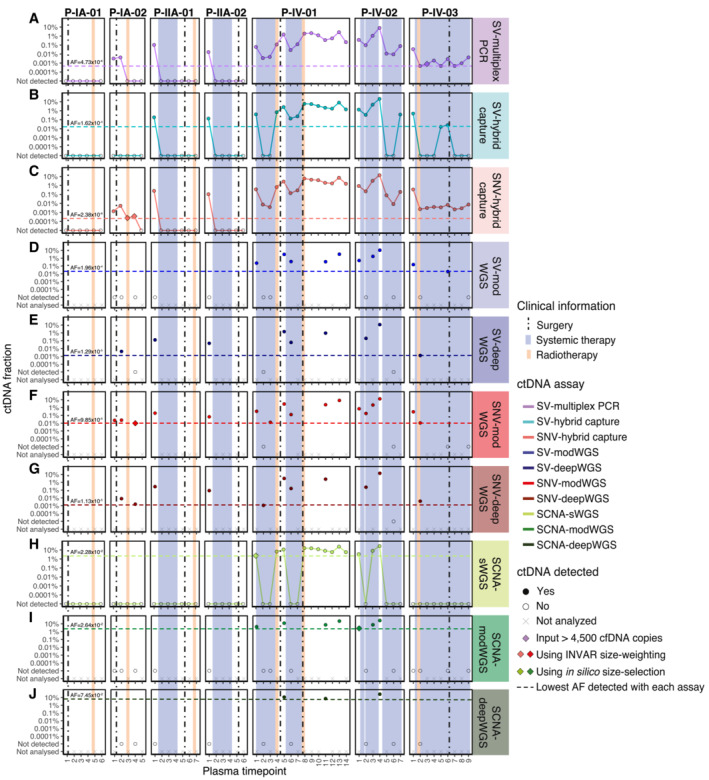
ctDNA detection and fractions using the different ctDNA assays ctDNA fractions are plotted as an allele fraction for SV/SNV assays and as tumor fraction for SCNA assays. (A–C) Targeted assays and (H) shallow WGS (sWGS, mean depth 1.2×) were performed in all plasma samples (*n* = 54), (D, F, I) moderately deep WGS (modWGS, mean depth 20×) in a subset of 21 plasma samples, and (E, G, J) deep WGS (deepWGS, mean depth 399×) in a subset of 12 samples (included also in modWGS). Plots (A–J) show the ctDNA fraction from each individual assay. (A) SV‐multiplex PCR assay: all detected plasma samples were assayed using up to 4,500 cell‐free DNA amplifiable copies except patient P‐IV‐03 plasma timepoint 3, which was detected after increasing the input amount to 27,000 cell‐free DNA copies; (B) SV‐hybrid capture assay; (C) SNV‐hybrid capture assay: patient P‐IA‐02 plasma timepoint 3 and timepoint 4 were detected only after applying INVAR size‐weighting feature; (D) SV‐modWGS assay; (E) SV‐deepWGS; (F) SNV‐modWGS: patient P‐IA‐02 plasma timepoint 4 was detected only after applying INVAR size‐weighting feature; (G) SNV‐deepWGS; (H) SCNA‐sWGS assay: patient P‐IV‐01 plasma timepoint 1 was detected only after *in silico* 90–150 bp size selection; (I) SCNA‐modWGS assay: patient P‐IV‐02 plasma timepoint 1 was detected only after *in silico* 90‐150 bp size selection; and (J) SCNA‐deepWGS.

As each of the patient‐specific SV‐multiplex PCR assays was shown to be linear and quantitative, experiments were performed to analyze cell‐free DNA extracted from 54 serial plasma samples collected from the seven breast cancer patients. Each patient‐specific assay was also performed in buffy coat from the same patient, plasma from a pool of five healthy donors, and water as a no‐template control (NTC). The number of reads in plasma samples from patients and controls before applying the filtering for detection are shown in Table EV8. The number of input copies was deliberately limited to induce a digital nature to the assay, therefore the majority of SV targets (defined as a given SV in a given replicate) had no reads. The overall median in all cases was therefore 0, so the value reported in Table EV8 is the median number of reads for all SV targets with any reads. Details on how data were analyzed, including filtering and the detection criteria used, can be found in Materials and Methods. Overall, ctDNA was detected in 34/54 (63%) longitudinal plasma samples using the patient‐specific SV‐multiplex PCR assays, and was not detected in any of the controls. A median of 72 positive reads (range 1–20,887 reads) was generated from the plasma cell‐free DNA samples. In patients with late‐stage (IV) disease, ctDNA was detected in 100% (30/30) of the plasma samples analyzed (Fig 3A, Tables EV9 and EV10). In patients with early‐stage (I–II) disease, ctDNA was detected in 4/24 (16%) of the samples, including 3 of the 4 baseline samples (all except P‐IA‐01) and the plasma samples collected on the same day before and after surgery from patient P‐IA‐02. The lowest AF observed was 0.00047% (4.7 parts per million, ppm) in P‐IV‐03 plasma timepoints 2, 5, and 7. This AF is at the theoretical limit of detection in this patient, with 1 SV detected in plasma out of a possible estimated 211,500 targets (47 SVs targeted × input of 4,500 copies). ctDNA detection for these samples is based on the detection of a single‐input mutant molecule and, therefore, the AF quantification is subject to stochastic nature of sampling and has a large range of uncertainty. Indeed, ctDNA was initially not detected in plasma timepoint 3 from this same patient when 4,500 amplifiable copies were assayed but detected when the same sample was re‐assayed using 27,000 copies, with an observed AF of 0.00079% (7.9 ppm, 10 SVs detected). For five other plasma samples, analysis was performed using an input of >4,500 amplifiable copies (9,000–36,000 copies), but ctDNA was not detected using these higher‐input amounts (Table EV10). In all plasma samples from healthy controls and buffy coats, 4,500 amplifiable copies were assayed.

#### SV‐hybrid capture and SNV‐hybrid capture assays

Sequencing libraries were prepared using SureSelect^XT HS^ (Agilent) from fragmented tumor DNA from the 7 breast cancer patients, matched fragmented buffy coat DNA, and plasma cell‐free DNA from 54 patient samples and 18 individual healthy donors. Hybrid capture was performed using the appropriate bait set for each patient. Hybrid capture libraries from plasma samples were sequenced to a mean unique depth of 471× (range 62× – 1,063×). Sequencing information showing the median paired reads generated from patients and control samples is shown in Table EV6.

##### SV‐hybrid capture analysis of serial plasma samples

Structural variants were analyzed in plasma from 54 patient samples and 18 healthy donors using SV‐hybrid capture (Table EV2), targeting between 30 and 153 per patient (409 in total). A median of 18 patient‐specific reads per SV (range 1–1,115 reads) were observed for the appropriate patients. The number of reads observed per SV in patient plasma samples and healthy controls before applying any filtering for detection can be found in Table EV11. Details on how data were analyzed and the detection criteria used can be found in Materials and Methods. With the SV‐hybrid capture assay, ctDNA was detected in 20/30 (67%) of the samples from patients with late‐stage disease, including plasma timepoint 1 from all three patients (Fig 3B, Tables EV9 and EV10). In samples from patients with early‐stage disease, ctDNA was detected only in timepoint 1 from P‐IIA‐01 and P‐IIA‐02. ctDNA was not detected in any of the healthy plasma controls. The lowest allele fraction obtained with this assay was at 0.016% AF for P‐IV‐03 plasma timepoint 5. This is the patient with the highest number of SVs targeted (*n* = 153) and, therefore, had the lowest theoretical limit of detection.

##### SNV‐hybrid capture analysis of serial plasma samples

SNV‐hybrid capture was performed in tumor and buffy coat from the seven patients, in plasma from 54 patient samples, and in plasma from 18 healthy donors. Analyses were performed using INVAR (Wan *et al*, 2020). A subset of 1,347–7,491 SNVs (unique to a single patient) were targeted per patient in plasma, matched tumor, and matched buffy coat (Tables EV2 and EV4), and all SNVs were tested in the 18 healthy controls. Sequencing data from potentially duplicated reads were collapsed using minimal family sizes of 2, 3, and 5 for comparison purposes (Table EV12). Family sizes indicate the minimum number of sequencing reads required to generate a consensus sequence. A consensus sequence represents a family of paired reads with the same fragment end position and same UMI and allows the reconstruction of original biological molecules by identifying and removing PCR and sequencing errors (University of Michigan, 2016). Specificity was measured on all unrelated samples (healthy samples and samples from patients where the particular mutations were not expected to be present). A specificity of >95% was used to classify the patient‐specific samples and limit the false‐positive rate. The specificity increased when increasing the family size, although as expected the total number of molecules retained for analysis decreased. With family size 2, ctDNA was detected in 34/54 (63%) samples at INVAR specificity > 95%. With family sizes 3 and 5, ctDNA was detected in 36/54 (67%) of samples at INVAR specificity > 95% as two samples with specificities of 92% and 91% in family size 2 had specificities exceeding 95% in family sizes 3 and 5 (Table EV12). The data we present in method comparisons are from read collapsing using family size 3, as this resulted in high retention of molecules for analysis and high specificity. At the patient‐specific loci, in the seven tumors, the median allele fractions and interquartile ranges were P‐IA‐01: 30.0% (18.9–38.0%); P‐IA‐02: 14.3% (9.1–18.9%); P‐IIA‐01: 20.0% (11.5–26.2%); P‐IIA‐02: 31.9% (16.5–39.1%); P‐IV‐01: 16.8% (11.7–25.3%); P‐IV‐02: 36.8% (16.1–44.6%) and P‐IV‐03: 23.8% (15.7–36.9%). In each buffy coat, the median observed allele fraction was zero (the upper bound of the interquartile ranges in all cases was less than 0.5%).

Of the 4,500 amplifiable copies of cell‐free DNA (measured by dPCR using the *RPP30_97bp* amplicon) used as input for patient samples and controls, the number of unique copies sequenced per locus was between 103 and 757. Given that the dPCR assay quantifies single strands of DNA, this indicates that a mean of 15% starting amplifiable DNA molecules produced useable sequencing results after library preparation, capture, and sequencing (Table EV13). Across all the targeted loci, this equated to a mean of 1.3 million molecules analyzed. INVAR analysis includes a patient‐specific outlier suppression filter (which removes signal from one locus if not consistent with the distribution of the remaining loci) designed for low AF samples. To avoid underestimation of the AF in stage IV patients where the AF was expected to be high (P‐IV‐01, P‐IV‐02, and P‐IV‐03), the calculation of the AF excluded this filter when the number of loci detected before its application was > 25%. This included the 14 samples with the highest AF (Table EV13).

At a specificity > 95%, ctDNA was detected in all samples from patients with late‐stage disease and 6/24 (25%) of samples from patients with early‐stage disease, including the baseline samples from 3/4 patients (all except P‐IA‐01) and samples collected before and after surgery from patient P‐IA‐02 (Fig 3C, Tables EV9 and EV10). Table EV14 shows the number of mutant reads initially observed (when the bam file is summarized by pileup) prior to INVAR analysis, in comparison to the number observed after INVAR filtering. INVAR uses custom error suppression and is designed to enrich tumor‐specific reads and remove noisy loci where mutant reads are less likely to originate from the tumor. After INVAR filtering, healthy or unrelated samples had low signal (median of 0 mutant reads, range 0–10), whereas detected samples had a median of 808 mutant reads (range 8–118,739) across the samples analyzed. ctDNA was detected in P‐IA‐02 plasma timepoints 3 and 4 after applying the INVAR size‐weighting feature, which gives greater weight to molecules with a size range similar to the size distribution of ctDNA and, therefore, boosts the signal in samples with low levels of ctDNA. Overall ctDNA was detected in 36/54 (67%) samples using the SNV‐hybrid capture assay and INVAR. The sample detected with the lowest AF was P‐IA‐02 plasma timepoint 3 at 0.00024% AF (2.4 ppm).

### Tumor‐informed assays: targeted analysis of known SVs and SNVs using whole‐genome sequencing

Given the cost of sequencing, WGS at various depths of sequencing (range 15× – 505×) was performed on a subset of 21 plasma samples from patients and 4 individual healthy donors. To reduce variation due to sequencing depth differences, data were randomly downsampled to 600 M reads (mean sequencing depth 20×) for the modWGS assay, including 21 plasma samples from patients and 4 from individual healthy donors. Of these, 12 patient plasma samples were originally sequenced to a mean unique depth of 399× (range 267× – 505×), prior to downsampling, for the deepWGS assay. The number of samples assayed and paired reads generated with each assay can be found in Table EV6.

#### SV analysis of serial plasma samples using SV‐modWGS and SV‐deepWGS

A total of 409 structural variants (between 30 and 153 per patient) were analyzed with SV‐modWGS and SV‐deepWGS (Table EV2). In the SV‐modWGS assay, 21 plasma samples from patients and 4 from individual healthy donors were analyzed. Given that the analyzed SVs are patient‐specific mutations but genome‐wide sequencing was performed on all samples, we were able to additionally leverage the use of other patients' samples as controls. The number of reads observed per SV in plasma samples and healthy controls before applying any filtering for detection can be found in Table EV11. Details on how data were analyzed and the detection criteria used can be found in Materials and Methods. No reads were observed in any healthy donor plasma sample but one non‐specific read from a different patient was observed when analyzing SVs from Patients P‐IV‐01 and P‐IV‐02, which may be due to index hopping of sample barcodes within the same sequencing lane. For the AF calculations, all patient‐specific reads were considered. ctDNA was only detected in samples from patients with late‐stage disease, with an overall detection rate of 48% (10/21) (Fig 3D, Tables EV9 and EV10). ctDNA was not detected in early‐stage samples or healthy controls. The lowest allele fraction detected was at 0.02% AF in P‐IV‐03 plasma timepoint 6. This patient had the highest number of SVs targeted (*n* = 153) and, therefore, the lowest theoretical limit of detection.

In the SV‐deepWGS assay, 12 plasma samples from patients were run in different sequencing lanes and no non‐specific reads were observed in any sample (Table EV11). Here, we were also able to leverage the use of other patient samples as controls. Patient‐specific SVs were observed in 75% (9/12) of the samples, including 3/4 samples from patients with early‐stage disease and 6/8 samples from patients with late‐stage disease (Fig 3E, Tables EV9 and EV10). The lowest AF detected with this assay was 0.0013% in P‐IV‐03 plasma timepoint 2, corresponding to the patient with the highest number of SVs targeted (*n* = 153).

#### SNV analysis of serial plasma samples using SNV‐modWGS and SNV‐deepWGS

All patient‐specific SNVs identified in WGS of the tumor and buffy coat and passing the filters of Mutect2 or Strelka were analyzed in SNV‐modWGS and SNV‐deepWGS data using the INVAR pipeline (Wan *et al*, 2020), analyzing between 4,153 and 16,015 SNVs per patient (Tables EV2 and EV4). In the SNV‐modWGS assay, 21 plasma samples from early‐ and late‐stage patients and 4 from individual healthy donors were analyzed. In addition to using the data from healthy donors, other patients' samples could be used as controls at loci where they were known to not be mutated. At a specificity > 95%, ctDNA was detected in all samples analyzed from early‐stage patients (5/5) with an overall detection rate of 81% (17/21), (Fig 3F, Tables EV9 and EV10). Using the likelihood ratio scores, thresholds were selected (as described in Statistical Considerations in Materials and Methods). Patient P‐IA‐02 plasma sample timepoint 4 was detected at the required specificity only after size selection (specificity of 94.3% before size selection and 97.2% after size selection) and is the sample with the lowest AF detected at 0.0098% AF. In modWGS for all the cases, there are more mutant reads in detected samples than undetected, unrelated, and healthy samples prior to INVAR analysis (Table EV14). After INVAR filtering, the number of mutant reads in healthy and unrelated samples was low (median 0 and range 0–28 reads), whereas detected samples had a median of 366 (range 12–21,546) mutant reads across the samples analyzed.

In the SNV‐deepWGS assay, 12 plasma samples from early‐ and late‐stage patients were analyzed. In these assays, ctDNA was detected in all samples analyzed from early‐stage patients (4/4) with an overall detection of 92% (11/12) (Fig 3G, Tables EV9 and EV10). The sample with the lowest AF detected was P‐IV‐01 plasma timepoint 2 with ctDNA detected at AF of 0.0011%. The number of mutant reads before and after INVAR filtering is shown in Table EV14. The majority of noisy loci were removed by INVAR filters such that after INVAR, unrelated samples had low signal (median of 0 mutant reads and range 0–63), whereas detected samples had a median of 1,002 mutant reads (range 26–103,000) across the samples analyzed.

### Tumor‐naïve assays: whole‐genome sequencing assays evaluating SCNAs

In the sWGS assays, whole‐genome libraries prepared from the seven tumor and buffy coat samples, 54 patient plasma samples, and 18 healthy donor samples were sequenced to a mean 1.2× coverage (range 0.6× to 1.8×; Table EV6). In all of the SCNA assays, ichorCNA (Adalsteinsson *et al*, 2017) was used to evaluate the presence of copy‐number aberrations and estimate the tumor fraction in plasma (from patients and healthy donors), tumor tissue and buffy coat samples, using the recommended detection threshold of 3% (Fig 3H–J). To enrich the signal from tumor‐derived ctDNA, *in silico* size selection of 90‐150 bp fragments was also performed in plasma samples, as previously described (Mouliere *et al*, 2018). As size selection increases the ichorCNA value for all samples, size‐selected samples were classified as detected if SCNAs were observed and were concordant with those in sWGS of tumor tissue of the appropriate patient, while reporting the tumor fraction calculated prior to size selection (Table EV10). No SCNAs were detected in healthy control samples before size selection and all had lower ichorCNA values than in the patient samples detected after size selection. The median and range of observed ichorCNA values are shown for patient and healthy plasma samples in Table EV15.

#### SCNA‐sWGS of tumor, buffy coat, and serial plasma samples before and after *in silico* size selection

SCNA‐sWGS assays were performed on tumor and buffy coat from the seven patients as well as in plasma from 54 samples from patients and 18 healthy donors.

**Figure EV2:**
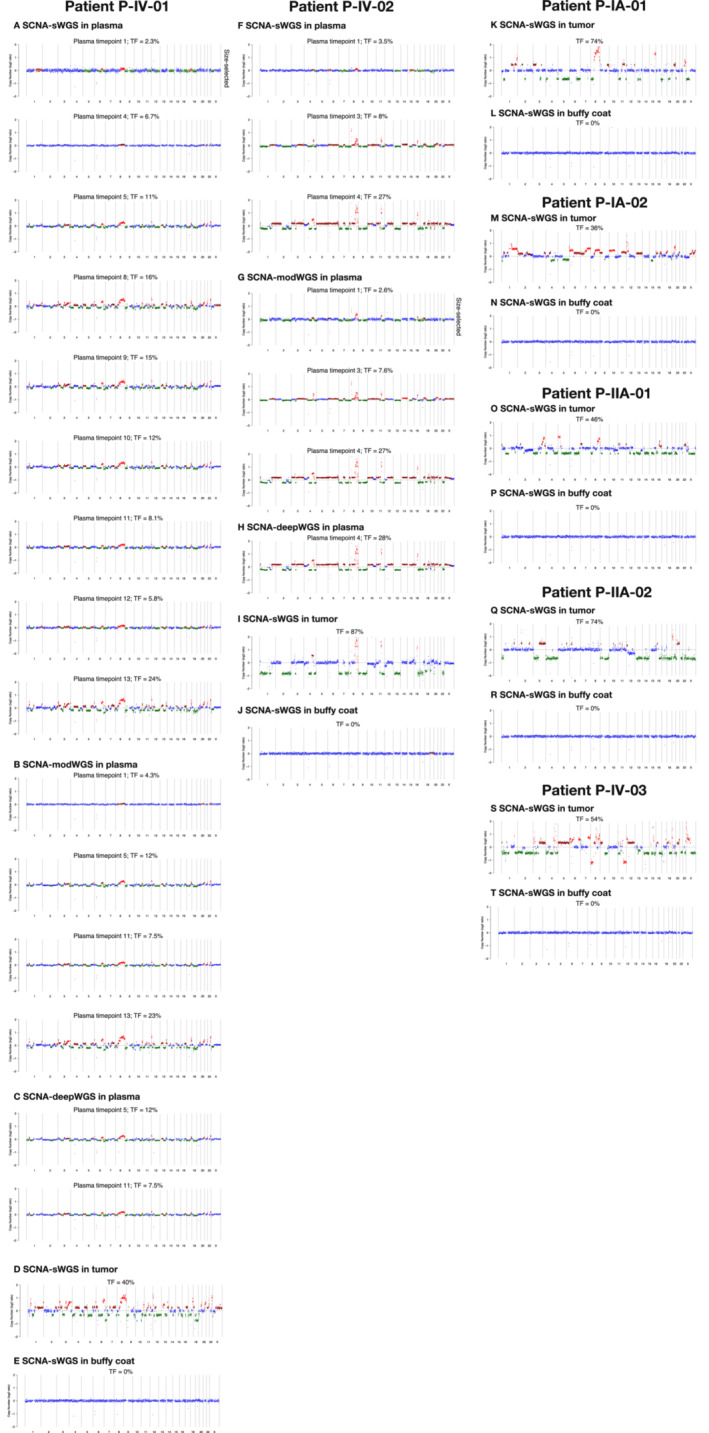
SCNA plots and tumor fraction (TF) observed with the SCNA assays in plasma All plasma samples with ctDNA detected either before or after size selection with SCNA‐sWGS, modWGS, and deepWGS are plotted as well as tumor and buffy coat from the seven patients. Plots and tumor fraction (TF) plotted before size selection for all samples except those marked as “size‐selected”; in those samples, the plot corresponds to the SCNAs observed after size selection, while the tumor fraction is that generated before size selection. Tumor fraction estimated from ichorCNA. (A–E) SCNA plots from P‐IV‐01, (A) plasma samples detected with SCNA‐sWGS, (B) plasma samples detected with SCNA‐modWGS, (C) plasma samples detected with SCNA‐deepWGS, (D) sWGS of tumor tissue, and (E) sWGS of buffy coat. (F–J) SCNA plots from P‐IV‐02, (F) plasma samples detected with sWGS, (G) plasma samples detected with SCNA‐modWGS, (H) plasma samples detected with SCNA‐deepWGS, (I) sWGS of tumor tissue, and (J) sWGS of buffy coat. For both patients, all plasma samples detected have similar alterations between different plasma samples from the same patient tested with the different SCNA assays as well as with the tumor tissue. (K–T) sWGS of tumor and buffy coat from the remaining five patients with no plasma sample detected by the analysis of SCNAs.

Before *in silico* size‐selection, ctDNA was detected with SCNA‐sWGS in 12/54 plasma samples, and the lowest estimated tumor fraction observed in a plasma sample with ctDNA detected was 3.5% (P‐IV‐02 plasma timepoint 1) (Fig 3H). ctDNA was not detected in any of the healthy plasma controls. Specific SCNAs could be observed in all samples with ctDNA detected and the profiles were concordant between different plasma samples and the tissue from the same patient (Fig EV2). SCNA aberrations could be observed in all seven tumor samples, with a tumor fraction between 36% and 87%. All buffy coats showed flat profiles and were classified as undetected by ichorCNA (all with tumor fraction = 0). Due to its GC richness, false positives are expected in chromosome 19 and can be observed on the buffy coat of P‐IV‐02 (Straver *et al*, 2014).

**Figure 4:**
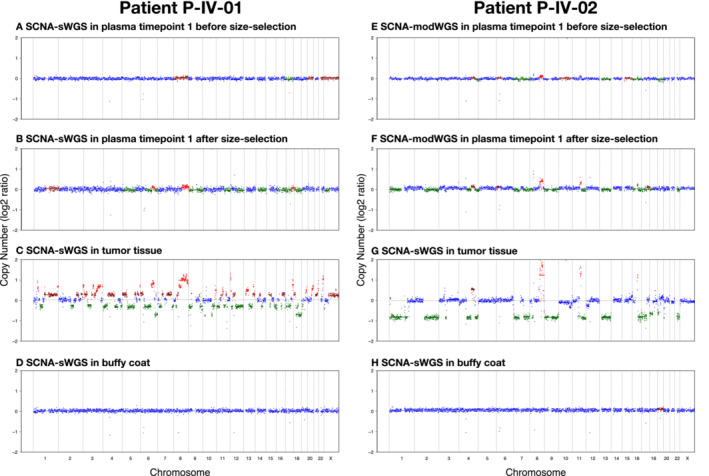
SCNA signal enrichment after *in silico* 90–150 bp size selection in patients P‐IV‐01 and P‐IV‐02 (A–D) Patient P‐IV‐01: When analyzed with SCNA‐sWGS, (A) P‐IV‐01 plasma timepoint 1 shows a flat profile before *in silico* size selection and a tumor fraction of 2.3%, that is below the ichorCNA recommended cut‐off of 3%, while (B) after 90–150 bp size selection, copy‐number changes can be observed. These copy numbers match those observed (C) in tumor and are not present in (D) matched buffy coat. (E–H) P‐IV‐02: (E) When analyzed with SCNA‐modWGS, P‐IV‐02 plasma timepoint 1 has a tumor fraction below the ichorCNA recommended cut‐off of 3% (tumor fraction of 2.6%) and is, therefore, classified as undetected even though small copy‐number changes can be observed. (F) Following *in silico* size selection, those changes become more apparent. The copy‐number aberrations match those observed (G) in tumor and are not present in (H) matched buffy coat.

After *in silico* size selection, SCNAs were observed in one additional plasma sample (patient P‐IV‐01 plasma timepoint 1, Fig 4A–D) that had a non‐size‐selected tumor fraction below the threshold of detection for ichorCNA. The SCNAs observed in this plasma sample following *in silico* size‐selection‐matched copy‐number profiles observed in tumor tissue of the same patient (Fig EV2). Overall, ctDNA was detected with SCNA‐sWGS in 13/54 plasma samples, all from patients with late‐stage disease (Fig 3H, Tables EV9 and EV10). All detected samples had higher ichorCNA values than non‐detected samples and healthy controls. The median and range of values are shown in Table EV15.

#### SCNA‐modWGS analysis of serial plasma samples before and after *in silico* size selection

SCNA‐modWGS was performed in 21 plasma samples from patients and 4 from individual healthy donors. Before *in silico* size selection, ctDNA was detected using SCNA‐modWGS in 6/21 samples, and the lowest estimated tumor fraction observed was at 4.3% in P‐IV‐01 plasma timepoint 1. ctDNA was not detected in any of the healthy plasma controls. The SCNAs observed were concordant between different plasma samples and tumor tissue from the same patient in the different SCNA assays (Fig EV2). After *in silico* size selection, SCNAs were observed in one additional plasma sample (P‐IV‐02 plasma timepoint 1) (Figs 3I and 4E–H). Overall, ctDNA was detected with SCNA‐modWGS in 7/21 samples (Fig 3I, Tables EV9 and EV10). All detected samples had higher ichorCNA values than non‐detected samples and healthy controls, and the median and range of values are shown in Table EV15.

#### SCNA‐deepWGS analysis of serial plasma samples before and after *in silico* size selection

In the SCNA‐deepWGS, 12 plasma samples from patients were analyzed. ctDNA was detected in 3/12 samples prior to *in silico* size selection, with the lowest tumor fraction observed at 7.5% in P‐IV‐01 plasma timepoint 11 (Fig 3J, Tables EV9 and EV10). SCNAs were observed in all these samples and were concordant between different plasma samples and tumor tissue from the same patient in the different SCNA assays (Fig EV2). After *in silico* size‐selection SCNAs were not observed in any additional sample. The median and range of values are shown in Table EV15.

### Comparison of the different ctDNA assays

**Figure 5:**
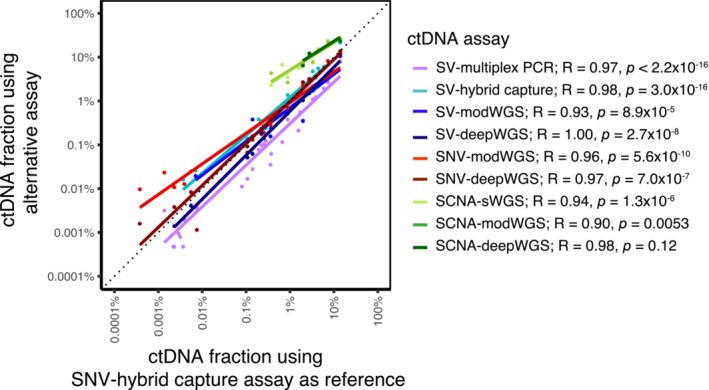
Correlation of the ctDNA fractions calculated with all assays performed Allele fractions plotted for SV/SNV assays, and tumor fractions for SCNA assays. The SNV‐hybrid capture assay was used as reference (x axis), with the other assays plotted on a common y axis. Colored lines correspond to the linear regression fits per assay. The Pearson's correlation coefficient estimates and *P*‐values of the corresponding tests of associations are also indicated. Spearman rank correlations led to the same conclusions (range *R* = 0.98 to *R* = 1.00).

**Figure EV3:**
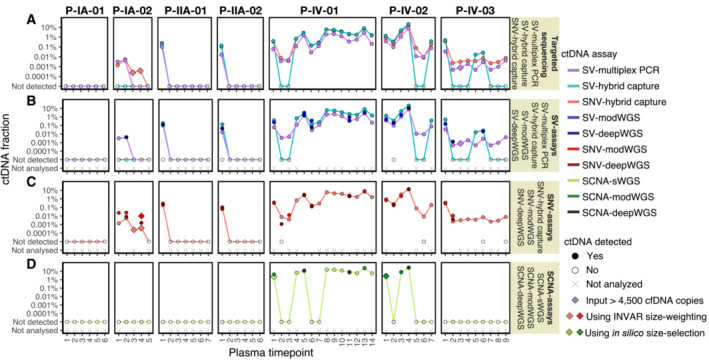
ctDNA detection and fractions using the different ctDNA assays ctDNA fractions are plotted as an allele fraction for SV/SNV assays and as tumor fraction for SCNA assays. Plots (A–D) show comparisons of the detection and ctDNA fraction obtained with different assays. (A) Comparison of targeted sequencing assays (SV‐multiplex PCR, SV‐hybrid capture, and SNV‐hybrid capture); (B) Comparison of all assays targeting SVs (SV‐multiplex PCR, SV‐hybrid capture, SV‐modWGS, and SV‐deepWGS); (C) Comparison of all assays targeting SNVs (SNV‐hybrid capture, SNV‐modWGS, and SNV‐deepWGS); and (D) Comparison of all assays evaluating SCNAs (SCNA‐sWGS, SCNA‐modWGS, and SCNA‐deepWGS). The individual plots and number of samples assayed are shown in Fig 3.

**Figure EV4:**
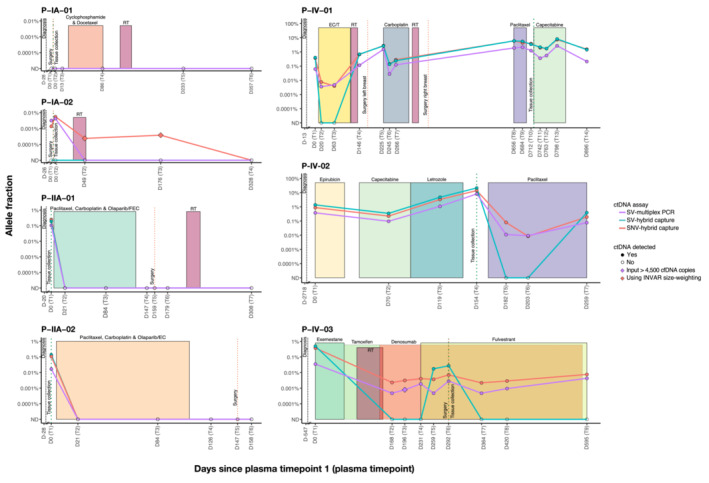
Clinical information and detection of ctDNA in each patient using targeted ctDNA assays The shaded boxes represent different treatment periods and the vertical dotted lines mark the time of diagnosis (black), the time of surgery where appropriate (dark orange), and the time of tissue collection for whole‐genome sequencing (dark green). Days indicated on the x axis refer to the number of days before or after the collection of the first plasma sample. Two samples were taken at day 0 (D0) in stage IA patients (P‐IA‐01 and P‐IA‐02), before (T1) and after surgery (T2). Individual plots showing the allele fraction from every ctDNA assay can be found in Fig 3. RT: radiotherapy; FEC: 5 fluorouracil (5FU), epirubicin, and cyclophosphamide; EC: epirubicin and cyclophosphamide; /T followed by docetaxel; ND: Not detected.

Figure EV3 and Table EV9 summarize and compare the performance of the assays described above. In samples where ctDNA was detected, similar ctDNA dynamics were observed using the different assays (Fig EV3A–D). The subset of most sensitive assays is also shown in Fig EV4, and demonstrates the relationship between ctDNA dynamics and clinical treatment over time. To further compare the assays, linear regression was performed using Pearson correlation, with SNV‐hybrid capture used as the reference assay as this had the highest number of samples detected. Results shown in Fig 5 highlight the strong correlation between the AFs and tumor fractions estimated using the different assays. SCNA‐modWGS (*R* = 0.90, *P* = 0.053) and SCNA‐deepWGS (*R* = 0.98, *P* = 0.12) showed a similar trend but did not reach significance level of 0.05, likely due to the low number of samples detected (7/21 and 3/12, respectively).

#### Comparison of the targeted sequencing assays

In our comparison, SNV‐hybrid capture (Fig 3C) was the most sensitive assay, detecting ctDNA in 67% (36/54) of the samples, down to an AF of 0.00024% (2.4 ppm). The second most sensitive assay was SV‐multiplex PCR (Fig 3A), detecting ctDNA in 63% (34/54) of the samples down to an AF of 0.00047% (4.7 ppm). SNV‐hybrid capture and SV‐multiplex PCR detected ctDNA in the same plasma samples, with the additional two samples with ctDNA detected by hybrid capture when applying INVAR size‐weighting features. The least‐sensitive targeted sequencing assay was SV‐hybrid capture (Fig 3B), detecting ctDNA in 41% (22/54) of the samples down to an AF of 0.016% (a detection threshold two orders of magnitude less sensitive than with SV‐multiplex PCR and SNV‐hybrid capture). In our cohort, in all the samples in which ctDNA was detected by targeting SVs with any assay, ctDNA was also detected by SNV‐hybrid capture (Fig EV3A, Table EV9).

#### Comparison of all assays targeting SVs

We next compared the detection of SVs using WGS assays (i.e., SV‐modWGS, Fig 3D; and SV‐deepWGS, Fig 3E) to using targeted sequencing assays (SV‐multiplex PCR and SV‐hybrid capture, Fig EV3C). Using SV‐hybrid capture, ctDNA was detected in three more samples than with SV‐modWGS (Table EV16). Both SV‐hybrid capture and SV‐modWGS targeted the same number of SVs (30–153), while the mean sequencing coverage depth in SV‐modWGS was on average ~ 30 times lower (mean 20× compared to 584× in this subset of 21 samples). Using SV‐multiplex PCR, ctDNA was detected in 10 additional samples compared to SV‐modWGS (Table EV16).

Using SV‐deepWGS, ctDNA was detected in 9/12 samples (Table EV16). Of these, ctDNA was detected in eight by SV‐hybrid capture. For both assays, the same number of SVs were targeted (30–153) and the sequencing depth was on average ~ 1.5 times lower in the SV‐deepWGS assay (399× compared to 591× for this subset of 12 samples). The detection of ctDNA in the additional sample (P‐IV‐03 plasma timepoint 2) using SV‐deepWGS resulted from the identification of only two SVs and therefore potentially subject to stochastic effects. Using the SV‐multiplex PCR, ctDNA was detected in two additional samples compared to SV‐deepWGS (P‐IV‐01 plasma timepoint 2 and P‐IV‐02 plasma timepoint 6).

#### Comparison of assays targeting SNVs

Table EV17 shows a comparison of the SNVs identified by SNV‐hybrid capture (Fig 3C), SNV‐modWGS (Fig 3F), and SNV‐deepWGS (Fig 3G). Due to restrictions in the size of the bait‐set design, the SNV‐hybrid capture assay targeted a lower number of loci than both WGS assays (1,347–7,491 vs. 4,153–16,015, respectively). The depth of sequencing was higher for SNV‐hybrid capture (mean 584× in the subset of 21 samples analyzed by SNV‐modWGS) than for SNV‐modWGS (mean 20×) and SNV‐deepWGS (mean 399×), and the number of informative sequencing reads analyzed by SNV‐hybrid capture (mean 1.4 M reads) was on average 8.6 times higher than with SNV‐modWGS (mean 162 K reads) and almost half that with SNV‐deepWGS (mean 2.4 M reads). In all 21 samples analyzed by modWGS, ctDNA was detected by SNV‐hybrid capture, while ctDNA was detected by SNV‐modWGS in 17/21 and by SNV‐deepWGS in 11/12. In both SNV‐hybrid capture and SNV‐modWGS, P‐IA‐02 plasma timepoint 4 was only detected at the required specificity after applying INVAR's size‐weighting feature (Fig EV3C).

#### Comparison of assays evaluating SCNAs

The assays targeting SCNAs (SCNA‐sWGS, Fig 3G; SCNA‐modWGS, Fig 3H; and SCNA‐deepWGS, Fig 3J) had the lowest detection rates (Table EV9). ctDNA was not detected in any sample from early‐stage patients using any of these assays. As previously indicated, *in silico* size selection enabled detection of ctDNA in additional plasma samples (Fig 4). All samples in which ctDNA was detected by either SCNA‐modWGS or SCNA‐deepWGS also had ctDNA detected with SCNA‐sWGS and showed similar tumor fractions (Table EV10, Fig EV3D). All SCNA detected in plasma were concordant with those observed in sWGS of tumor tissue of the appropriate patient.

## Discussion

Here, we present the development and comparative performance of different tumor‐informed and tumor‐naïve assays for the quantification of ctDNA in serial plasma samples collected from patients with stage IA–IV breast cancer before and during treatment. Tumor‐informed assays (SV‐multiplex PCR, SV‐hybrid capture, SNV‐hybrid capture, SV‐modWGS, SV‐deepWGS, SNV‐modWGS, and SNV‐deepWGS) leveraged patient‐specific mutations identified by whole genome‐sequencing of tumor and germline DNA to analyze SVs and SNVs in cell‐free DNA extracted from plasma samples. In the tumor‐naïve assays, which did not require prior knowledge of the tumor‐specific mutations, we analyzed somatic copy‐number aberrations at different sequencing depths of WGS data (SCNA‐sWGS, SCNA‐modWGS, and SCNA‐deepWGS). To our knowledge, this is the first study comparing the performance of multiple different tumor‐informed and tumor‐naïve assays for ctDNA detection and quantification of SVs, SNVs, and SCNAs in the same serial plasma samples from breast cancer patients. Results demonstrate that similar ctDNA dynamics were observed in samples where ctDNA was detected using the different assays, with the AFs being strongly correlated. No ctDNA was detected using any assay in analysis of plasma samples from patient P‐IA‐01, a patient with stage IA disease at diagnosis. This patient subsequently showed no evidence of disease relapse within 3 years after collection of the last plasma timepoint.

Assay sensitivity is governed by different factors, including the number of DNA molecules analyzed (which can be increased by analyzing larger volumes of blood plasma), the efficiency of the assay, sequencing depth, and the number of mutations targeted. Recent studies have shown that it is possible to increase the sensitivity of ctDNA detection by analyzing a greater number of mutations, compared to the analysis of single‐mutant loci (Newman *et al*, 2016; Abbosh *et al*, 2017; McDonald *et al*, 2019; Wan *et al*, 2020). In this study, we used whole‐genome sequencing of tumor and germline DNA to identify mutations which were used to design personalized assays for ctDNA analysis. This study has the advantage of using whole‐genome rather than whole‐exome sequencing, enabling identification of a greater number of somatic mutations for ctDNA analysis (4,153–16,015 SNVs and 31–156 SVs per patient).

We have shown that tumor‐informed SNV‐hybrid capture assays targeting thousands of patient‐specific mutations have the highest sensitivity for detecting low levels of ctDNA, with detection down to 0.00024% AF (2.4 ppm). This is approximately 1.5 orders of magnitude lower than that observed using the Signatera assay which has a limit of detection of 0.01% AF when targeting 16 patient‐specific clonal SNVs and indels (Coombes *et al*, 2019). SNV‐hybrid capture leverages INVAR analysis features for boosting ctDNA signal in low AF samples; two samples were only detected after applying the INVAR size‐weighting feature. With the exception of these two samples, SV‐multiplex PCR and SNV‐hybrid capture detected ctDNA in exactly the same plasma samples, demonstrating that both assays have high sensitivity and could detect ctDNA showing similar dynamics. The two samples where ctDNA was not detected using SV‐multiplex PCR can be explained by the very low AFs measured only by size‐weighted INVAR analysis (patient P‐IA‐02 plasma timepoint 3 at 0.00024% AF; plasma timepoint 4 at 0.00038% AF). The SV‐multiplex PCR assay for this patient targeted 21 SVs (the lowest number of mutations targeted in all the assays developed) and, given the 4,500 amplifiable copies used as cell‐free DNA input, the theoretical limit of detection is 0.0011% AF. Detecting ctDNA in these samples using the multiplex PCR assay would in theory require an input of 20,000 and 12,600 amplifiable copies, respectively, which was not available for analysis. Alternatively, if the cell‐free DNA input was fixed at 4,500 amplifiable copies, 93 and 59 patient‐specific SVs would theoretically be required to detect ctDNA in these samples using SV‐multiplex PCR.

As structural variants have been relatively under‐studied in ctDNA research, we wanted to assess the performance of using SV‐multiplex PCR and SV‐hybrid capture to determine their relative sensitivity for analysis of ctDNA. Chromosomal rearrangements are often clonal, formed in early tumor development and “passenger” mutations, not subject to positive or negative selection pressures (Wang *et al*, 2014). Furthermore, analysis of gross genomic rearrangements in plasma cell‐free DNA has the potential advantage of reduced background signal, compared to single‐nucleotide sequence changes which may also arise from PCR and sequencing errors. Here, we have been able to demonstrate that, despite targeting fewer structural variants, the SV‐multiplex PCR approach used was more sensitive than the SV‐hybrid capture assay, detecting ctDNA in 63% compared to 41% of patient plasma samples. The lowest AF detected using SV‐multiplex PCR was 1.5 orders of magnitude lower than that detected by SV‐hybrid capture (0.00047% AF vs. 0.016% AF). The lower detection rate using SV‐hybrid capture is likely to be due to a greater loss of DNA molecules during the library preparation stages (66% to 95% loss in this study; Table EV13) as a result of inefficient adaptor ligation, compared to multiplex PCR amplification using primers to more efficiently incorporate adaptor sequences. That said, hybrid capture has the advantage that it can assay more SVs or mutations than multiplex PCR, which is limited in the number of targets that can be multiplexed together due to unpredictable interactions between amplicons, particularly for structural rearrangements which may contain highly repetitive sequences. Furthermore, compared to hybrid capture, SV‐multiplex PCR has potentially higher upfront costs to fully optimize and validate the assays.

Identification of *de novo* patient‐specific SVs is still challenging and needs more research. However, we have been able to demonstrate the ability to target selected large‐span SVs associated with copy‐number aberrations and detect ctDNA with high sensitivity. Given that SNV‐based multiplex PCR ctDNA assays are already starting to prove effective in the detection of MRD in a clinical setting (Abbosh *et al*, 2017; Coombes *et al*, 2019; Gale *et al*, 2022), the incorporation of patient‐specific SV assays may have added advantages and should be further investigated as a clinical tool for detection and monitoring of ctDNA.

While the costs of whole‐genome sequencing currently limit its routine clinical use, recent advances in sequencing technology indicate that the promise of a $100 genome may become a reality, making WGS more readily affordable in the future (Almogy *et al*, 2022; Illumina Press Release, 2022; Rusinek *et al*, 2022; Ultima Genomics Press Release, 2022). As a proof of principle to assess the performance of analysis of plasma cell‐free DNA to identify both SVs and SNVs using different depths of whole‐genome sequencing, we performed modWGS and deepWGS on a subset of 21 and 12 samples, respectively. However, given the current costs of deep sequencing, only samples with ctDNA previously detected with targeted sequencing were selected for analysis, resulting in overall inflation of the fraction of samples detected compared to other methods. In the analysis of SVs, due to the lower depth of coverage of modWGS, detection of some samples relied on the identification of only one to two patient‐specific SVs so their AF quantification may potentially be less precise. Still, these results suggest that patient‐specific SVs can be identified using WGS to ~ 20× coverage. SV‐deepWGS detected one additional sample compared to SV‐hybrid capture, although both methods had a similar average sequencing depth. This sample was detected based on one single read in two different SVs and may have been missed using capture due to the stochastic effects of sampling bias.

In the analysis of SNVs, modWGS and deepWGS targeted a higher number of SNVs than SNV‐hybrid capture due to restrictions in the size of the bait‐set design used for hybrid capture. Despite this, SNV‐hybrid capture was the most sensitive method, detecting ctDNA in four more samples than SNV‐modWGS. With SNV‐deepWGS, ctDNA was detected in P‐IA‐02 plasma timepoint 4, while with SNV‐hybrid capture and SNV‐modWGS, ctDNA was only detected after applying INVAR's size‐weighting feature. This further supports the use of fragment size to enhance the sensitivity of assays for ctDNA detection, such as INVAR, by enabling detection of ctDNA that may have otherwise been considered background noise (Mouliere *et al*, 2018; Wan *et al*, 2020).

Our findings show that, as expected, the least sensitive assays were those analyzing copy‐number aberrations using either sWGS, modWGS or deepWGS. We showed that SCNA signal could be enhanced using *in silico* size selection to boost tumor‐specific signal (Fig 4) as previously shown by our group (Mouliere *et al*, 2018), but increasing the depth of sequencing did not appear to increase sensitivity. SCNAs were not detected in any samples from early‐stage patients using any of the WGS assays. In plasma from patients with advanced cancer, similar tumor fractions were observed using whole‐genome sequencing at the three different depths of coverage (Table EV10). Close to the ichorCNA lower limit of detection, detection of ctDNA appeared to be stochastic.

In this study, when using a tumor‐naïve approach, we only analyzed somatic copy‐number aberrations. Additional approaches that could be used include the analysis of genome‐wide methylation signatures (Liu *et al*, 2020) or different biological features, such as fragment length, the nucleotide context at the ends of fragments, relative coverage at nucleosome positions, and open chromatin regions. These biological features could potentially be incorporated into machine learning algorithms to improve the sensitivity of diagnostic assays (Chandrananda *et al*, 2015; Lo *et al*, 2021; Mathios *et al*, 2021). Zviran et al. developed MRDetect and demonstrated the use of 35× WGS and genome‐wide mutational integration to detect tumor fractions to < 0.001%, with increased detection close to 0.0001% AF using 120× WGS data of tumor:normal synthetic admixtures (Zviran *et al*, 2020). Additional sensitive approaches have recently been developed including PhasED‐Seq (Kurtz *et al*, 2021) and SaferSeqS (Cohen *et al*, 2021). PhasED‐Seq uses tumor WGS to first identify “phased variants” with two or more SNVs localized on the same DNA molecule, followed by plasma hybrid capture analysis to detect down to less than 1 part per million (0.000094% AF). SaferSeqS uses efficient tagging of both Watson and Crick strands followed by duplex sequencing to reduce the error rate by >100‐fold compared to alternative molecular barcoding approaches, enabling detection down to < 0.001% AF.

The main limitation of this proof‐of‐concept study is the low number of samples and patients analyzed. We were able to assay 54 longitudinal plasma samples from seven stage IA–IV patients undergoing treatment and 19 samples from healthy donors using up to 10 different assays. This enabled us to assess relative assay performance across a comprehensive range of ctDNA levels that would be expected in a large cohort, detecting ctDNA down to parts per million. This study should ideally be expanded to fully test the performance of different assays targeting the same number of SVs, SNVs, and SCNAs in ctDNA in a larger cohort of patients within specific clinical settings to assess their utility. Furthermore, the assays used were not developed for clinical diagnostic use, and need to undergo full analytical validation prior to clinical application to assess the relative sensitivity and specificity of each assay type for detection of different mutation classes.

Multiple studies are currently exploring the potential of using patient‐specific assays to identify patients at high risk of relapse, who may benefit from adjuvant therapy, or to de‐escalate treatment in patients with no residual ctDNA detected post‐treatment, thereby avoiding unnecessary side‐effects (Abbosh *et al*, 2017; Gale *et al*, 2022; Tie *et al*, 2022). Currently, the development of tumor‐informed assays is challenging in the clinical setting given the high cost, the mandatory requirement for tumor and germline samples, and the time required for patient‐specific assay development. In the future, it is expected that tumor sequencing will become more affordable and routine in the clinic, enabling tumor‐informed assays to be more readily developed. A tumor‐naïve assay would be ideally used in the clinical setting given they avoid some of the operational challenges associated with accessing tumor and developing assays within a clinically‐relevant timeframe. However, tumor‐naïve assays are currently not sufficiently sensitive for detection of low‐burden disease. Further research is required to improve the sensitivity of tumor‐naïve assays to the required levels of sensitivity, through incorporation of additional features or biomarkers (Cohen *et al*, 2018), as this would have obvious benefits to optimize treatment regimes in patients with earlier‐stage disease.

Understanding the relative performance of different ctDNA assays to detect levels of tumor burden in blood enables the most appropriate assay to be selected for each specific intended use. To our knowledge, this study provides the most comprehensive analysis to date comparing the performance of different tumor‐informed and tumor‐naïve ctDNA assays targeting different genomic alterations using multiple cutting‐edge methods in the same patient cohort. sWGS has significant advantages as it can be performed within a relatively short turnaround time, has relatively low costs as it only requires low‐coverage sequencing, and does not need prior analysis of the tumor, thereby making it attractive as a clinical diagnostic assay. However, due to its relatively low sensitivity, the assay may currently only be used to identify copy‐number changes in late‐stage patients who have relatively high levels of tumor burden. Incorporating fragment size features into the analysis may potentially improve the sensitivity of detection. On the other hand, SNV‐hybrid capture, targeting thousands of mutations, and SV‐multiplex PCR, targeting tens of structural rearrangements, appear to have high sensitivity down to a few parts per million, and may be most applicable for use in patients with early‐stage and low‐burden disease where assay sensitivity is of critical importance.

## Materials and Methods

### Aims, design, and settings of the study

The aim of this study was to develop and compare different assays for the sensitive detection and quantification of ctDNA in plasma samples from patients with early‐ and late‐stage breast cancer targeting different genomic alterations (SNVs, SVs, and SCNAs). Using matched sequencing data from deep WGS of the tumor tissue and buffy coat of every patient, tumor‐informed assays were developed to target patient‐specific SVs and SNVs. Tumor‐naïve assays (i.e., without prior knowledge of the tumor tissue) were also developed to evaluate SCNAs.

The tumor‐informed assays included the evaluation of SVs and SNVs by targeted sequencing (SV‐multiplex PCR, SV‐hybrid capture, and SNV‐hybrid capture), and by WGS at various sequencing depths (SV‐modWGS, SV‐deepWGS, SNV‐modWGS, and SNV‐deepWGS). The tumor‐naïve assays relied on the evaluation of SCNAs by WGS at various sequencing depths (SCNA‐sWGS, SCNA‐modWGS, and SCNA‐deepWGS). The same plasma samples were analyzed by the different assays (*n* = 54 samples), although due to the costs of sequencing, modWGS and deepWGS were performed in a subset of these (*n* = 21 and *n* = 12 samples, respectively). modWGS includes a set of patients sequenced at various depths and then downsampled to 600 M reads; this set includes the 12 samples analyzed by deepWGS (prior to downsampling).

Following the optimization of each assay, we compared (a) their performance in the detection of ctDNA, (b) the quantification of the ctDNA fraction in each sample (AF for assays detecting mutant alleles or tumor fraction for assays measuring SCNAs), and (c) the correlation of the ctDNA fractions calculated using the different assays.

### Patients and samples

Stage I‐IV breast cancer patients were recruited to the Personalized Breast Cancer Programme (PBCP), and tumor biopsies and matched normal (buffy coat) samples were collected for whole‐genome sequencing (WGS). Serial plasma samples (*n* = 54) were collected prior to and during treatment from seven patients: two with stage IA disease, two with stage IIA, and three with stage IV. Plasma from healthy donors was purchased from BioIVT (19 plasma samples, 18 from individual healthy donors plus 1 pool from 5 individuals).

### Ethics approval and consent to participate

This work was approved by the East of England (Cambridge Central) Health Research Authority [REC IDs: 16/EE/0100; 15/NW/0926; 15/NW/0994; 07/Q0106/63]. The PBCP study is registered in the NIHR Clinical Research Network database (Registration number 39296). Informed written consent was obtained from all participants. The experiments conformed to the principles set out in the WMA Declaration of Helsinki and the Department of Health and Human Services Belmont Report.

### Statistical considerations

In this comparative analysis, no sample size estimations or randomization were performed. Patient samples were de‐identified to researchers performing experiments. Experiments were not performed blind, as researchers were developing patient‐specific assays, so needed to know which sample came from each patient. No analyzed samples were omitted from report. Samples were omitted from analysis where insufficient material was available. Given the limited amount of patient material, it was not possible to perform independent replicate experiments. Linear regressions (with the response and predictor on the log10 scale) were used to analyze the relationship between observed and expected allele fractions. *R*‐squared estimates were used to quantify the level of association, and test of significance of the regression slope parameter was performed by means of Wald *t*‐tests within the R linear model. The correlation between the AFs calculated using the different assays was assessed using Pearson's coefficient as well as the more robust Spearman rank correlation estimator.

For INVAR analysis, a likelihood ratio was generated for each sample through aggregation of signal across all patient‐specific loci in that sample using the generalized likelihood ratio test, as previously described (Wan *et al*, 2020). The patient‐specific loci were identified by prior tumor and buffy coat sequencing (as described in the section on Identification of Patient‐Specific SVs and SNVs, below). Loci adjacent (+/− 10 bp) to each locus of interest were used to determine the trinucleotide background error rate. Samples were classified as “ctDNA detected” or “ctDNA not detected” based on comparison of likelihood ratios between patient‐matched plasma samples and plasma samples from other patients, where these loci are not expected to have a mutation signal (as the mutations were selected as private to a given patient based on tumor WGS). For the SNV‐hybrid capture dataset, such unrelated samples could only be used when their sequencing libraries were captured using the same bait set. For SNV‐modWGS and SNV‐deepWGS, any unrelated sample could be leveraged. To act as a control, the unrelated samples were required to have AF < 1%. The threshold for the likelihood ratio was determined by 10‐fold iterative resampling with replacement. Data from healthy individuals separately underwent the same steps to establish the specificity value for each dataset.

### Genomic and cell‐free DNA extraction and quantification

Tumor needle core biopsies (14‐18G) were collected at diagnosis (stage IIA patients), surgery (stage IA patients), or from metastatic sites (stage IV patients) and snap‐frozen in liquid nitrogen within 1 h of collection. Cellularity and tumor content were assessed by hematoxylin and eosin (H&E) staining of one to two (6–8 μm) frozen sections. Genomic DNA (gDNA) was extracted from one to two core biopsies using the AllPrep DNA/RNA Mini Kit (Qiagen). After dissolving the OCT (optimal cutting temperature compound) with 1 ml of distilled water, the tissue was transferred to a new tube with 5 mm stainless steel beads and 600 μl of RLT Plus buffer and homogenized twice in 1‐min rounds at 25 Hz with a TissueLyser II (Qiagen).

Peripheral whole blood from patients was collected into K_3_EDTA tubes and processed within 1 h of venipuncture by double centrifugation: 1,600 *g* for 10 min for separation of plasma and buffy coat followed by centrifugation of plasma supernatant at 16,000 *g* for 10 min. Plasma and buffy coat from patients were stored at −80°C until DNA extraction. Germline gDNA from buffy coat of patients and cell‐free DNA from plasma of patients and controls were extracted using a QIASymphony SP automated workstation (Qiagen). gDNA was extracted from 200 μl of buffy coat using the DSP DNA mini kit (Qiagen). Cell‐free DNA from patients and healthy donors was purified from 2 to 4.1 ml of plasma using the QIAsymphony DSP Circulating DNA kit (Qiagen). A non‐human spike‐in control was added to the lysis buffer during cell‐free DNA extraction to assess extraction efficiency, as previously described (Tsui *et al*, 2018). gDNA and cell‐free DNA were stored at −80°C until use.

Buffy coat and tumor gDNA were quantified using the Qubit dsDNA HS or BR Assay Kits (Thermo Fisher Scientific) and the Spectramax® Gemini XPS (Molecular Devices). The number of amplifiable copies of plasma cell‐free DNA was determined using digital PCR on a Biomark HD (Fluidigm), using a 65 bp assay targeting the *RPP30* locus as previously described (Tsui *et al*, 2018). The number of amplifiable copies is defined as the number of single‐stranded fragments of DNA amplified by the assay primers (Parkinson *et al*, 2016).

### Identification of patient‐specific SVs and SNVs

Whole‐genome sequencing (WGS) was performed by Illumina (Granta Park, Great Abington, Cambridge) on tumor (median 116× coverage) and matched germline gDNA (median 39× coverage) from buffy coat to identify tumor‐specific structural variants (SVs) and single nucleotide variants (SNVs). Libraries were prepared using an input of 600 ng DNA with the TruSeq® DNA PCR‐Free Library Preparation kit (Illumina) and sequenced using 150 bp paired‐end sequencing on a HiSeqX (Illumina). Buffy coat libraries were sequenced in one lane and tumor libraries in three lanes distributed across different flow cells.

High‐confidence, patient‐specific SVs were selected by filtering and matching calls to copy‐number steps. Tumor and matched buffy coat reads were aligned using Isaac Genome Alignment Software v. 03.16.02.19 (Raczy *et al*, 2013) to the reference genome GRCh38 with decoy sequences and SVs were called by Illumina using Manta (Chen *et al*, 2016). The following were discarded: SVs mapped to unassembled or mitochondrial chromosomes; calls with Manta's SomaticScore < 31; and possible mismapping or polymorphism artifacts. These include breaks adjacent to gaps or overlapping simple repeats of > 100 bp; and recurrent breakpoints. Recurrent breakpoints were found by pooling SVs from 150 (these and additional) cases and identifying clusters of rearrangement breakpoints, either SVs spanning > 100 kb with breakpoints in different tumors separated by < 2 kb or all SVs with breakpoints separated by < 200 bp. High‐confidence SVs were identified as inter‐chromosomal and large (> 100 kb span) intra‐chromosomal SVs whose breakpoints were within 10 kb of copy‐number steps identified by Canvas (Roller *et al*, 2016).

Patient‐specific SNVs were called using two different pipelines: an Illumina pipeline using Strelka (Saunders *et al*, 2012) and an in‐house pipeline using Mutect2 (Broad Institute, 2022). For the Illumina pipeline, SNVs were called from the aligned sequences described above; for the in‐house pipeline, tumor and matched buffy coat reads were aligned with BWA‐MEM to the same reference genome followed by sorting with SAMtools (Li, 2003; Li *et al*, 2009) and marking duplicate reads using Picard tools (Broad Institute, 2019). SNVs within low‐sequencing complexity regions (including satellite repeats, simple repeats, homopolymers, and tandem duplications) were excluded. Variants present within the gnomAD germline resource (Karczewski *et al*, 2020) or in a pooled panel of normals (compiled by calling germline variants from WGS data derived from buffy coat of a cohort of 200 breast cancer patients) were removed. The following hard filters were applied to retain high‐quality variants: mapping quality >50, and tumor and germline sequencing coverage > 25. Strelka‐specific filters were used including SNV QSS score > 50. Mutect2 calls flagged as having a high OxoG artifact probability were also removed. Mutation co‐ordinates were lifted over from the GRCh38 assembly to hg19 using the LiftOver tool to facilitate panel design with SureDesign (Agilent) for the custom hybrid capture assay.

### Design of the different ctDNA assays, library preparation, and sequencing

#### Design of SV‐multiplex PCR assay, preparation of amplicon libraries, and sequencing

For the SV‐multiplex PCR assay, primers were designed surrounding the predicted breakpoints using Primer3Plus (Untergasser *et al*, 2007) for fragments of predicted genome between 82 and 144 bp to enable amplification of fragmented cell‐free DNA. Primers were preferentially selected in non‐repetitive regions with unique BLAT hits (https://genome.ucsc.edu) (Kent, 2002). The selected primer pairs were tested on tumor and matched germline DNA or whole‐genome libraries, in single‐plex and in a multiplex pool, followed by sequencing on a MiSeq (Illumina) using the PCR conditions detailed below. Primer pairs were discarded (a) if, after single‐plex PCR, reads from the targeted SV sequence were observed in germline DNA, (b) if reads were not observed in patient tumor DNA samples, or (c) if when running in multiplex, a high number of reads with a forward or reverse primer from a different primer pair were observed. To improve the assay efficiency, the concentration of some primers (either individually or as a pair) was doubled if a low number of reads was observed after sequencing when compared to the other primer pairs in the same pool. All patient‐specific primer pools included primers to amplify a 97 bp amplicon of *RPP30* (*RPP30_97bp)* as an internal positive control.

To perform the SV‐multiplex PCR, each DNA sample was divided and analyzed in several wells. Each patient‐specific assay was optimized and tested on serial dilutions of fragmented tumor DNA prior to the analysis of relevant patient plasma samples. Tumor and buffy coat gDNA were fragmented to ~150 bp using an S220 focused ultrasonicator (Covaris), followed by quantification by digital PCR on a Biomark HD (Fluidigm) and assessment of their size profile on a D1000 ScreenTape (Agilent). A first round of tumor dilution series was performed to assess the optimal PCR conditions and to estimate the tumor allele fraction (AF). Then, tumor DNA from all patients was diluted to an approximate AF of 10% and the dilution series repeated. Tumor dilutions were performed by diluting tumor DNA in buffy‐coat DNA from the same patient. Using the Poisson approximation, we estimated the input amplifiable copies needed per dilution based on the expected AF. The input DNA ranged from 30 to 4,500 amplifiable copies (0.1–15 ng). No template control (NTC; water) and neat fragmented buffy coat (4,500 input copies) were used as negative controls. Tumor dilutions were prepared at the following AFs: 10% (sample divided into three wells, total of 30 copies tested), 1% (3 wells, 90 copies), 0.1% (3 wells, 900 copies), 0.01%, 0.003%, 0.001%, and 0.0004% (5 wells per dilution, total of 4,500 copies in each dilution).

SV‐multiplex PCR assays included two rounds of amplification. In the first round of PCR, target sequences were amplified using a pool of patient‐specific primers with a common adapter. Ten microliters PCR reactions were performed containing 50 nM (or 100 nM for those with doubled concentration) of each forward and reverse target‐specific primer from a patient‐specific pool and the required input DNA amplifiable copies. The master mix contained 1 × FastStart High Fidelity Enzyme Buffer, 4.5 mM MgCl_2_, 0.5% DMSO, 0.05 U/μl of FastStart High Fidelity Enzyme Blend (FastStart™ High Fidelity PCR System, Roche), and 0.2 mM dNTPs (Deoxynucleotide (dNTP) Solution Mix, New England BioLabs). Reactions were subjected to amplification (95°C for 10 min; 35 cycles of 95°C for 15 s, 60°C for 30 s, and 72°C for 1 min; 72°C for 1 min). Five microliters of amplified product was transferred to a new plate with 2 μl of ExoSAP‐IT™ PCR Product Cleanup Reagent (Thermo Fisher Scientific) and incubated at 37°C for 15 min to remove excess primers and nucleotides, inactivated at 80°C for 15 min, then diluted 1/70 in nuclease‐free water. In a second round of PCR, Illumina sequencing adaptors and single‐index 10 bp barcodes were added in a 10 μl reaction containing 1 μl of diluted product from the first‐round PCR. Ten cycles of PCR were performed, using identical cycling conditions as the first‐round PCR, except that the barcoding primers were at a final concentration of 400 nM. A subset of samples was run on a D1000 ScreenTape (Agilent) to assess PCR performance. PCR products were pooled at equal volumes and run on a Pippin HT (Sage Science) to remove the adapter dimers, selecting DNA sized between 185 and 280 bp on a 2% agarose gel cassette.

After assessing the performance of the SV‐multiplex PCR assay on the tumor dilution series, 54 longitudinal plasma samples from patients were analyzed with their correspondent patient‐specific primer pool. All primer pools were also tested in replicate NTC and replicate plasma from healthy donors (a pool of five individuals) to control for PCR contamination and off‐target amplification, respectively. The number of replicates of the negative controls (NTC and healthy controls) was at least twice the number of replicates tested for each plasma sample. Amplicon libraries were initially generated using a DNA input ranging from 51 to 4,500 amplifiable copies. To increase the sensitivity of detection, five plasma samples where ctDNA was not detected using 4,500 input copies were assayed a second time using a higher input (up to 36,000 copies). Pools of 48–116 plasma libraries were sequenced using 150 bp paired‐end sequencing on a MiSeq V3 flow cell (Illumina) incorporating 15% spiked‐in PhiX Control v3 (Illumina) to increase library diversity.

#### Whole‐genome library preparation for sWGS, modWGS, and deepWGS assays

Whole‐genome libraries were generated from tumor and buffy coat from each of the seven patients, 54 longitudinal plasma samples from patients, and 18 individual healthy donors' plasma samples (10 of them prepared in triplicate generating 38 healthy donor libraries). Fifteen nanograms of tumor and buffy coat gDNA were fragmented prior to library preparation with the SureSelect Enzymatic Fragmentation Kit (Agilent). Libraries were prepared from the fragmented tumor or buffy coat gDNA (15 ng) or 4,500 amplifiable copies (~15 ng) of cell‐free DNA except for P‐IV‐01 plasma timepoints 8 and 9 in which 3,744 and 4,200 amplifiable copies were used, respectively. Libraries were prepared with the SureSelect^XT HS^ Reagent Kit (Agilent) using 11–15 amplification cycles. This kit includes unique‐molecular identifiers (UMI) and single indexing. All libraries were sequenced using 150 bp paired‐end sequencing to retain fragment‐size information. For the shallow WGS (sWGS) assays, libraries from all plasma samples from patients and healthy donors were sequenced by pooling 19–24 libraries per lane on a HiSeq 4000 (Illumina). Tumor and buffy coat libraries were pooled and sequenced in a NovaSeq 6000 S1 flowcell (Illumina) and resulting data were downsampled to 9 million reads. For the moderately deep WGS (modWGS) assays, two subsets of samples were included: (a) 9 libraries (including 5 plasma samples from patients and 4 from healthy donors) sequenced across two lanes of a NovaSeq 6000 S4 flowcell (Illumina) and (b) 12 plasma samples from patients that were sequenced on a NovaSeq 6000, running each sample in three lanes of different S4 flow cells, a strategy designed to prevent any risk of index hopping. Both subsets were downsampled using Picard tool PositionBasedDownsampleSam to the lowest number of reads of any sample, that is, to 600 M reads. Prior to downsampling, the 12 patient plasma samples (set b) were analyzed for the deep WGS assay (deepWGS).

#### Design of SV‐ and SNV‐hybrid capture assays, capture, and sequencing

Three custom hybrid‐capture bait sets were designed using SureDesign (Agilent), incorporating tumor‐specific mutations from the two stage IA patients (bait set IA), two stage IIA patients (bait set IIA), and three stage IV patients (bait set IV). Patient‐specific SVs, SNVs, indels, and a set of commonly mutated genes in breast cancer (hotspots from *AKT1* and exonic sequences from *TP53*, *MAP3K1*, *PTEN*, *ESR1*, *PIK3CA*, and *GATA3*) were included in the bait‐sets' design, but only SVs and SNVs were analyzed in this study. For SVs, 6× tiling was used (incorporating 10 probes per SV, with six baits spanning each breakpoint) with maximum performance boosting. Baits for SVs were designed against the GRCh38 reference coordinates (Table EV3). For SNVs, baits were designed for a subset of the SNVs identified by tumor WGS, and 2× tiling was used with balanced boosting. Bait set IA (27,493 baits, 2.345 Mbp) and bait set IIA (25,910 baits, 2.164 Mbp) incorporated all SNVs identified by tumor WGS and called by Mutect2, and were designed using least stringent masking. Due to a limitation in panel size, bait set IV (30,610 baits, 2.810 Mbp) incorporated the intersect of SNVs identified by both Mutect2 and Strelka, and baits were designed using moderately stringent masking.

Hybrid capture was performed in libraries from tumor and buffy coat of the 7 patients, 54 patient's plasma samples, and plasma from 18 individual healthy donors. Libraries were captured with the SureSelect^XT HS^ Target Enrichment System (Agilent) using 500 ng to 1,000 ng as input and 14 cycles of amplification. Libraries from plasma cell‐free DNA were captured in single plex. Libraries from tumor and buffy coat were pooled and captured in two‐plex (stage IA/IIA patients) or three‐plex (stage IV patients). The captured libraries were sequenced using 150 bp paired‐end sequencing on a NovaSeq 6,000 (Illumina). Tumor and buffy coat libraries were sequenced on an SP flow cell (14 libraries per lane) and plasma libraries on an S1 flow cell (20–24 libraries samples per lane). One pool of 21 plasma‐captured libraries was additionally sequenced in two lanes of a NovaSeq SP (Illumina).

### Detection and quantification of ctDNA using the different ctDNA assays

#### Evaluation of SVs in the SV‐multiplex PCR assay

To detect patient‐specific SVs in the SV‐multiplex PCR assay, a pipeline was developed that employs fuzzy matching functions to identify each primer pair and to compare the observed PCR amplicon sequences with the expected sequences spanning the breakpoint. This method iterates over each pair of fastq files and performs the following steps: (i) locating matching forward and reverse primer pairs in read 1 and read 2 separately, (ii) verifying the size of the target sequence and discarding any reads with short products (< 25 bp), (iii) extracting the amplicon sequence flanked by and including the primer sequences, (iv) comparing the observed and expected amplicon sequence, and (v) counting read pairs where both reads match to the same amplicon within a given edit distance. Each mismatch increases the edit distance by 1, as does an insertion or deletion of one base. An edit distance of up to 2 was allowed for each primer sequence match and a distance of up to 5 for the amplicon sequence. The use of fuzzy (as opposed to exact) matching enables recovery of reads with PCR errors that would not affect the specificity of SV detection. This approach requires that the amplicon matches across its entire length (considering the allowed edit distance) and that both reads in a pair match the same amplicon.

In the SV‐multiplex PCR assay, each patient‐specific SV was classified either as detected or as undetected, and the allele fraction (AF) for every sample was estimated based on the number of SVs detected across the wells in which that sample was divided. For an SV to be called as detected, we set a threshold for filtering out reads if they had less than two times the highest numbers of non‐specific reads observed in any negative control or an unrelated patient sample (i.e., ≤10 reads) because we reasoned that patient‐specific somatic SVs are only expected to be amplified using tumor DNA from the relevant patient. Low read counts (≤ 5) were unexpectedly observed in some of the negative controls and in unrelated patient samples, which may be due to low‐level index hopping, particularly in high AF samples. To test this, plasma samples were re‐pooled and re‐sequenced to a comparable depth but did not include any high AF samples. Ninety‐nine percent (71/72) of the samples did not have non‐specific reads, confirming that this was the likely cause and could in future be mitigated by a unique dual‐indexing strategy. For plasma samples, the AF was estimated as number of SVs detected across the wells / (sum of DNA input copies across the wells × number of patient‐specific SVs targeted). For samples with high AF, the probability of having more than one molecule with the same targeted SV on a particular well is higher, leading to an underestimation of the AF. Therefore, for the tumor dilution series, the Poisson approximation was used to estimate the AF as: [−(1/ number of DNA input copies in each well) * LN {1‐(number of SVs detected across the wells/(number of wells * number of patient‐specific SVs targeted))}].

#### Processing of sequencing reads from SureSelect^XT HS^ libraries

Illumina adapter sequences were removed with SurecallTrimmer (v.4.0.1) from the Agilent Genomics NextGen Toolkit (AGeNT), using a quality threshold of 5 for trimming and a minimum read length of 10% of the original read length after trimming. The 10 bp UMI was read as the second index and stored in a separate fastq file. The UMI was concatenated with read 1 and read 2 using Seqkit v.0.10.1 (Shen *et al*, 2016).

Sequencing reads were aligned to hg19 with BWA‐MEM v.0.7.17 for all assays except for the SV‐hybrid capture assay, in which the alignment was performed to a bespoke reference genome comprised from synthetic chromosomes (one per targeted SV, including 150 bp either side of the predicted breakpoint) and hs37d5 (GRCh37 with decoy sequences) to sequester non‐mutant reads (Li, 2003). Aligned reads were collapsed with Connor v.0.6.1 (University of Michigan, 2016), using a consensus alignment of 90% and various family sizes. Consensus sequences are then associated with the number of initial molecules harboring a specific alteration (SVs, SNVs, or SCNAs). The collapsed BAM files were sorted and indexed with SAMtools v.1.9 (Li *et al*, 2009). To remove the duplicates, data were collapsed to family size 1 except for the SV‐modWGS and SV‐deepWGS analyses, in which mutant reads were instead manually inspected to check for duplicates and for SNV‐hybrid capture, in which data were collapsed to family sizes 2, 3, and 5 for comparison purposes, selecting family size 3 for the data presented in the main text.

#### Evaluation of SVs in SV‐hybrid capture, SV‐modWGS, and SV‐deepWGS assays

In the SV‐hybrid capture assay, the number of reads per patient‐specific SV was assessed using bedtools coverage (Quinlan & Hall, 2010) as the number of reads spanning the breakpoint with a 100% match in the region covering 20 bp on either side of the center of the synthetic chromosome. Low‐level signal (≤ 4 reads per SV) was observed in negative controls or unrelated patients, so to circumvent issues with potential index hopping, AFs were calculated including all positive reads, and samples were classified as detected if (a) at least one targeted SV had more than two times the highest number of reads observed in negative controls or unrelated samples (i.e., more than 8 reads) or if (b) the number of targeted SVs with any number of reads was more than two times the highest number of targeted SVs with any reads observed in negative controls or unrelated samples (i.e., more than 20 SVs).

In the SV‐modWGS and SV‐deepWGS assays, a modified version of the fuzzy matching approach (used for the SV‐multiplex PCR assay) was used to identify patient‐specific SVs. Reads that had at least 10 bases clipped from the beginning or end of their alignment were extracted from the BAM files for further assessment as potential junction‐spanning sequences. Fuzzy string matching for the 20 bp flanking on each side of junctions was carried out using the aregexec function in R, allowing up to two mismatches in each flanking sequence. Those reads with matches for flanking sequences were then compared against the extended junction sequence using the R function adist, and retained if the edit distance was no more than 2.5% of the length of the read sequence. The UMI sequence at the beginning of each read was excluded from the flanking sequence search and edit distance calculation. The UMI tags were considered when tabulating the final counts of reads supporting each SV, excluding duplicate reads from the same DNA molecule arising from PCR amplification. For the AF calculations in SV‐modWGS and SV‐deepWGS assays, all patient‐specific reads were considered.

As these methods do not account for paired reads, in these assays, the AF was estimated as: number of reads spanning the breakpoint / (2 * average coverage depth of the sample * number of patient‐specific SVs targeted).

#### Evaluation of SNVs in SNV‐hybrid capture, SNV‐modWGS, and SNV‐deepWGS assays

INtegration of VAriant Reads (INVAR) (Wan *et al*, 2020) was used to evaluate patient‐specific SNVs from hybrid capture, modWGS, and deepWGS data. INVAR is an analysis pipeline that leverages custom error‐suppression and signal‐enrichment methods for sensitive detection of low levels of ctDNA. Custom error suppression includes (i) collapsing sequencing reads, (ii) requiring every mutation to be both in a forward and a reverse read, (iii) applying a locus noise filter, and (iv) applying a patient‐specific outlier suppression that removes the signal from one locus if not consistent with the distribution of the remaining loci. In order to exclude possible germline SNPs, only loci with AFs < 0.25 were used (based on the assumption that if a large number of loci are tested in a high ctDNA sample, the detection is supported by having many low AF loci with signal). Signal‐enrichment methods included assigning greater weight to (v) loci with higher AF observed in the tumor and to (vi) sequencing reads with a fragment size similar to the size distribution of ctDNA in the analyzed cohort.

In the SNV‐hybrid capture assay, a subset of SNVs (called by the intersect of both Mutect2 and Strelka variant callers and at loci covered by baits designed with moderately stringent masking) were analyzed to minimize background noise. INVAR was used to separately analyze the early‐stage (*n* = 11 stage IA and *n* = 13 stage IIA samples) and metastatic cohorts (*n* = 30 stage IV samples) due to their differences in ctDNA fragment‐size distribution. Default settings were used to run INVAR with the following exceptions: SLOP_BP = 20 (default 10, number of bp on either side of the target locus to assess the background error rate); Proportion_of_controls = 0.3 (default 0.1, proportion of non‐patient‐specific samples above which the blacklist loci need to have signal); and SIZE_COMBINED (path to sequencing reads to perform the size weighting) for the early‐stage cohort using the size distribution based on two other early‐stage cohorts (Mouliere *et al*, 2018) to increase the number of mutant fragments and to generate a proper fragment length distribution.

In the SNV‐modWGS and SNV‐deepWGS assay, all patient‐specific SNVs (identified in WGS of the tumor and passing the filters of Mutect2 or Strelka) were analyzed with the INVAR pipeline. Samples from early‐stage (*n* = 3 stage IA and *n* = 2 stage IIA samples for modWGS; *n* = 2 stage IA and *n* = 2 stage IIA samples for deepWGS) and metastatic patients (*n* = 16 stage IV samples for modWGS; *n* = 8 for deepWGS) were analyzed together due to the smaller sample set. INVAR was run using default settings except for MIN_DP = 1 (default 5, minimal depth to consider for mpileup).

In all these assays, the AF from INVAR analysis was estimated as an integrated mutant allele fraction (IMAF) as generated by the pipeline, which was optimized for the sensitive detection of low levels of ctDNA (Wan *et al*, 2020). The present cohort included several samples from metastatic patients and expected high levels of ctDNA. While INVAR is able to confidently detect ctDNA in these samples, the AF might be artificially decreased by inclusion of the patient‐specific outlier suppression for IMAF computation. Hence, if more than 25% of a patient's loci were detected before the application of the patient‐specific outlier suppression, the ctDNA fraction (namely the number of mutated reads divided by the total number of reads with the targeted loci) was computed by including all loci that passed all filters except the patient outlier suppression filter.

### Evaluation of SCNAs in the SCNA‐sWGS, SCNA‐modWGS, and SCNA‐deepWGS assays

SCNAs were assessed in all WGS assays (sWGS, modWGS, and deepWGS) with ichorCNA (Adalsteinsson *et al*, 2017). For the analysis of plasma samples, default controls and settings were used with the exception of (i) ‐‐normal “c(0.85,0.90,0.95,0.99,0.995,0.999),” (ii) ‐‐maxCN 4, and (iii) ‐‐estimateScPrevalence False. Tumor and buffy coat of each patient as positive and negative controls, respectively, were analyzed using the same settings as for plasma except that due to the known high tumor fraction, the possible starting normal value was reduced using the setting: (i) ‐‐normal “c(0.2,0.3,0.4,0.5,0.6,0.7,0.8,0.9)” and (ii) ‐‐maxCN 5. The tumor fraction was generated with ichorCNA, using the authors' suggested cut‐off for detection at 3%. In plasma samples where the tumor fraction was different from 0, *in silico* size selection was performed to select fragments between 90 and 150 bp to increase the sensitivity of detection of tumor‐derived DNA, as previously described (Mouliere *et al*, 2018). This increased the apparent tumor fraction of all samples, so size‐selected samples were classified as detected if SCNAs were observed when plotting the log2 ratio of the copy number while the reported tumor fraction was calculated before size selection.

## Author contributions

**Davina Gale:** Conceptualization; resources; data curation; formal analysis; supervision; validation; investigation; visualization; methodology; writing – original draft; writing – review and editing. **Angela Santonja:** Conceptualization; resources; data curation; software; formal analysis; validation; investigation; visualization; methodology; writing – original draft; writing – review and editing. **Wendy N Cooper:** Conceptualization; resources; data curation; formal analysis; validation; investigation; visualization; methodology; writing – original draft; writing – review and editing. **Matthew D Eldridge:** Data curation; software; formal analysis; writing – review and editing. **Paul AW Edwards:** Data curation; formal analysis; writing – review and editing. **James A Morris:** Data curation; software; formal analysis. **Abigail R Edwards:** Data curation; software; formal analysis; writing – review and editing. **Hui Zhao:** Data curation; software; formal analysis; writing – review and editing. **Katrin Heider:** Data curation; software; formal analysis. **Dominique‐Laurent Couturier:** Formal analysis; writing – review and editing. **Aadhitthya Vijayaraghavan:** Data curation; formal analysis; writing – review and editing. **Paulius Mennea:** Data curation; formal analysis; writing – review and editing. **Christopher G Smith:** Data curation. **Chris Boursnell:** Data curation; formal analysis. **Raquel Manzano García:** Formal analysis. **Oscar M Rueda:** Resources; formal analysis; writing – review and editing. **Emma Beddowes:** Resources. **Heather Biggs:** Resources; data curation. **Stephen‐John Sammut:** Resources; data curation; formal analysis; writing – review and editing. **Nitzan Rosenfeld:** Conceptualization; formal analysis; funding acquisition; writing – review and editing. **Carlos Caldas:** Conceptualization; resources; funding acquisition; writing – review and editing. **Jean E Abraham:** Conceptualization; resources; data curation; funding acquisition; writing – review and editing. **Emma‐Jane Ditter:** Investigation.

## Disclosure and competing interests statement

NR and DG are co‐founders of Inivata. CGS is a current employee of Inivata. DG, JAM, and KH are current employees of AstraZeneca. Inivata and AstraZeneca had no role in the conceptualization or design of the clinical study, statistical analysis, or decision to publish the manuscript. All other authors declare that they have no competing interests.

## Supporting information



Expanded View Figures PDF

Table EV1

Table EV2

Table EV3

Table EV4

Table EV5

Table EV6

Table EV7

Table EV8

Table EV9

Table EV10

Table EV11

Table EV12

Table EV13

Table EV14

Table EV15

Table EV16

Table EV17

PDF+

## Data Availability

The datasets and computer code produced in this study are available in the following databases:
Sequencing data: European Genome‐phenome archive EGAD00001008589 and EGAD00001006293 (https://ega‐archive.org/datasets/EGAD00001008589 and https://ega‐archive.org/datasets/EGAD00001006293). The correspondence between IDs of patients and plasma timepoints used in the paper and those in EGA are detailed in Table EV10. To obtain access, please email the corresponding authors and rosenfeld.labadmin@cruk.cam.ac.uk and complete a data access agreement with the University of Cambridge, which is required to respect patient confidentiality. INVAR code: Bitbucket (https://bitbucket.org/nrlab/invar/src/master/). Code for evaluating SVs: Github (https://github.com/nrlab‐CRUK/SV_detection).
