# Supplementary material for: Comparison of tumor-informed and tumor-naïve sequencing assays for ctDNA detection in breast cancer
Source: EMBO Mol Med. Author manuscript; Available in PMC 2023 Jun 8. (PMC10245040; doi:10.15252/emmm.202216505)
Supplement: EV Figures [file EMS175606-supplement-EV_Figures.zip › Figure EV1.pdf]

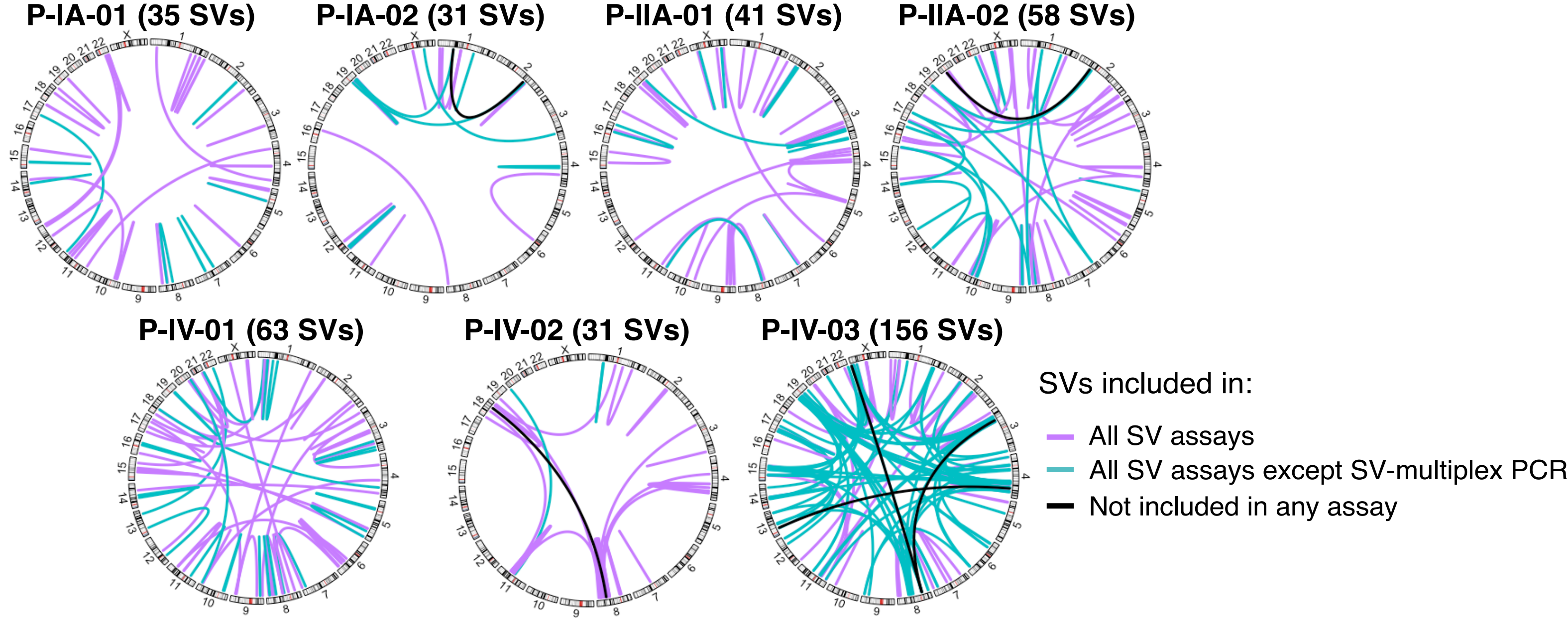
P-IA-01 (35 SVs)
P-IA-02 (31 SVs)
P-IIA-01 (41 SVs)
P-IIA-02 (58 SVs)
P-IV-01 (63 SVs)
P-IV-02 (31 SVs)
P-IV-03 (156 SVs)
SVs included in:
All SV assays
All SV assays except SV-multiplex PCR
Not included in any assay
