# Supplementary material for: Comparison of tumor-informed and tumor-naïve sequencing assays for ctDNA detection in breast cancer
Source: EMBO Mol Med. Author manuscript; Available in PMC 2023 Jun 8. (PMC10245040; doi:10.15252/emmm.202216505)
Supplement: EV Figures [file EMS175606-supplement-EV_Figures.zip › Figure EV2.pdf]

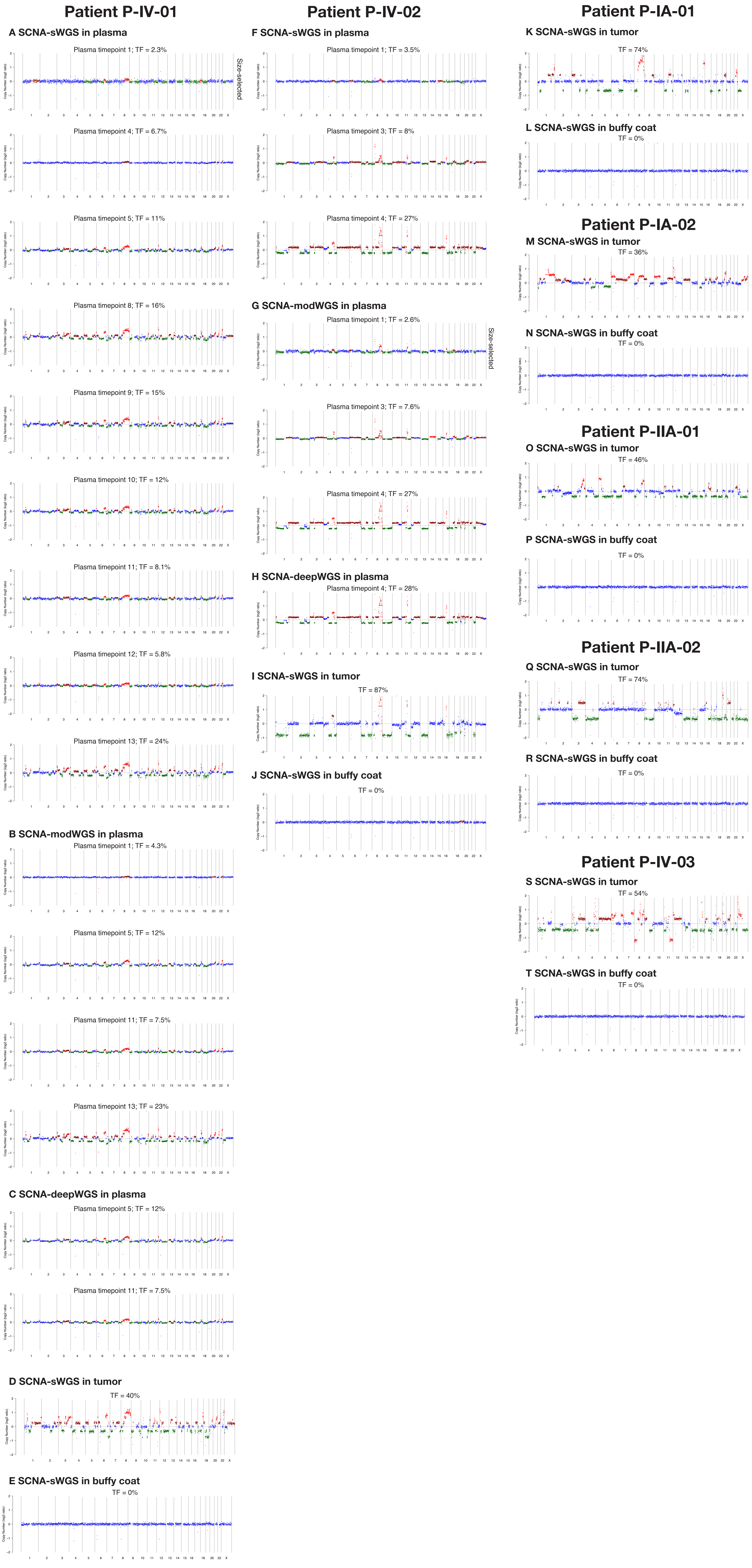
Patient P-IV-01
A SCNA-sWGS in plasma
Plasma timepoint 1; TF = 2.3%
Size-selected
Plasma timepoint 4; TF = 6.7%
Plasma timepoint 5; TF = 11%
Plasma timepoint 8; TF = 16%
Plasma timepoint 9; TF = 15%
Plasma timepoint 10; TF = 12%
Plasma timepoint 11; TF = 8.1%
Plasma timepoint 12; TF = 5.8%
Plasma timepoint 13; TF = 24%
B SCNA-modWGS in plasma
Plasma timepoint 1; TF = 4.3%
Plasma timepoint 5; TF = 12%
Plasma timepoint 11; TF = 7.5%
Plasma timepoint 13; TF = 23%
C SCNA-deepWGS in plasma
Plasma timepoint 5; TF = 12%
Plasma timepoint 11; TF = 7.5%
D SCNA-sWGS in tumor
TF = 40%
E SCNA-sWGS in buffy coat
TF = 0%
Patient P-IV-02
F SCNA-sWGS in plasma
Plasma timepoint 1; TF = 3.5%
Plasma timepoint 3; TF = 8%
Plasma timepoint 4; TF = 27%
G SCNA-modWGS in plasma
Plasma timepoint 1; TF = 2.6%
Size-selected
Plasma timepoint 3; TF = 7.6%
Plasma timepoint 4; TF = 27%
H SCNA-deepWGS in plasma
Plasma timepoint 4; TF = 28%
I SCNA-sWGS in tumor
TF = 87%
J SCNA-sWGS in buffy coat
TF = 0%
Patient P-IA-01
K SCNA-sWGS in tumor
TF = 74%
L SCNA-sWGS in buffy coat
TF = 0%
Patient P-IA-02
M SCNA-sWGS in tumor
TF = 36%
N SCNA-sWGS in buffy coat
TF = 0%
Patient P-IIA-01
O SCNA-sWGS in tumor
TF = 46%
P SCNA-sWGS in buffy coat
TF = 0%
Patient P-IIA-02
Q SCNA-sWGS in tumor
TF = 74%
R SCNA-sWGS in buffy coat
TF = 0%
Patient P-IV-03
S SCNA-sWGS in tumor
TF = 54%
T SCNA-sWGS in buffy coat
TF = 0%
