# Supplementary material for: Comparison of tumor-informed and tumor-naïve sequencing assays for ctDNA detection in breast cancer
Source: EMBO Mol Med. Author manuscript; Available in PMC 2023 Jun 8. (PMC10245040; doi:10.15252/emmm.202216505)
Supplement: EV Figures [file EMS175606-supplement-EV_Figures.zip › Figure EV4.pdf]

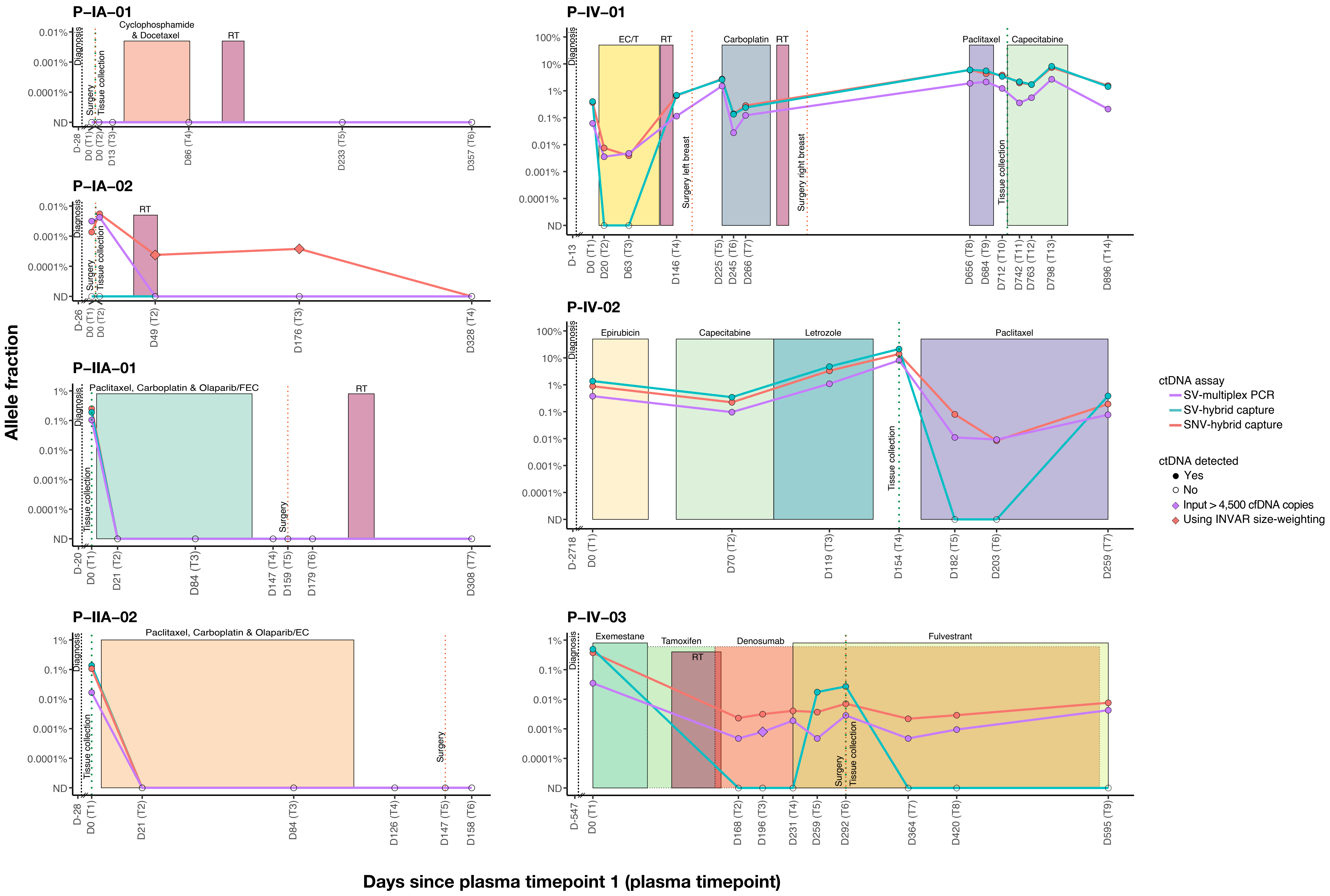

P–IA–01
Cyclophosphamide & Docetaxel
RT
Diagnosis
Surgery
Tissue collection
0.01%
0.001%
0.0001%
ND
D-28
D0 (T1)
D0 (T2)
D13 (T3)
D86 (T4)
D233 (T5)
D357 (T6)
P–IA–02
RT
D-26
D0 (T1)
D0 (T2)
D49 (T2)
D176 (T3)
D328 (T4)
P–IIA–01
Paclitaxel, Carboplatin & Olaparib/FEC
RT
1%
0.1%
D-20
D0 (T1)
D21 (T2)
D84 (T3)
D147 (T4)
D159 (T5)
D179 (T6)
D308 (T7)
P–IIA–02
Paclitaxel, Carboplatin & Olaparib/EC
D-28
D0 (T1)
D21 (T2)
D84 (T3)
D126 (T4)
D147 (T5)
D158 (T6)
P–IV–01
EC/T
RT
Carboplatin
RT
Paclitaxel
Capecitabine
Surgery left breast
Surgery right breast
100%
10%
D-13
D0 (T1)
D20 (T2)
D63 (T3)
D146 (T4)
D225 (T5)
D245 (T6)
D266 (T7)
D656 (T8)
D684 (T9)
D712 (T10)
D742 (T11)
D763 (T12)
D798 (T13)
D896 (T14)
P–IV–02
Epirubicin
Capecitabine
Letrozole
Paclitaxel
D-2718
D0 (T1)
D70 (T2)
D119 (T3)
D154 (T4)
D182 (T5)
D203 (T6)
D259 (T7)
P–IV–03
Exemestane
Tamoxifen
RT
Denosumab
Fulvestrant
D-547
D0 (T1)
D168 (T2)
D196 (T3)
D231 (T4)
D259 (T5)
D292 (T6)
D364 (T7)
D420 (T8)
D595 (T9)
Allele fraction
Days since plasma timepoint 1 (plasma timepoint)
ctDNA assay
SV-multiplex PCR
SV-hybrid capture
SNV-hybrid capture
ctDNA detected
Yes
No
Input > 4,500 cfDNA copies
Using INVAR size-weighting
