# Supplementary figures and images for: Comparison of tumor-informed and tumor-naïve sequencing assays for ctDNA detection in breast cancer
Source: EMBO Mol Med. Author manuscript; Available in PMC 2023 Jun 8. (PMC10245040; doi:10.15252/emmm.202216505)

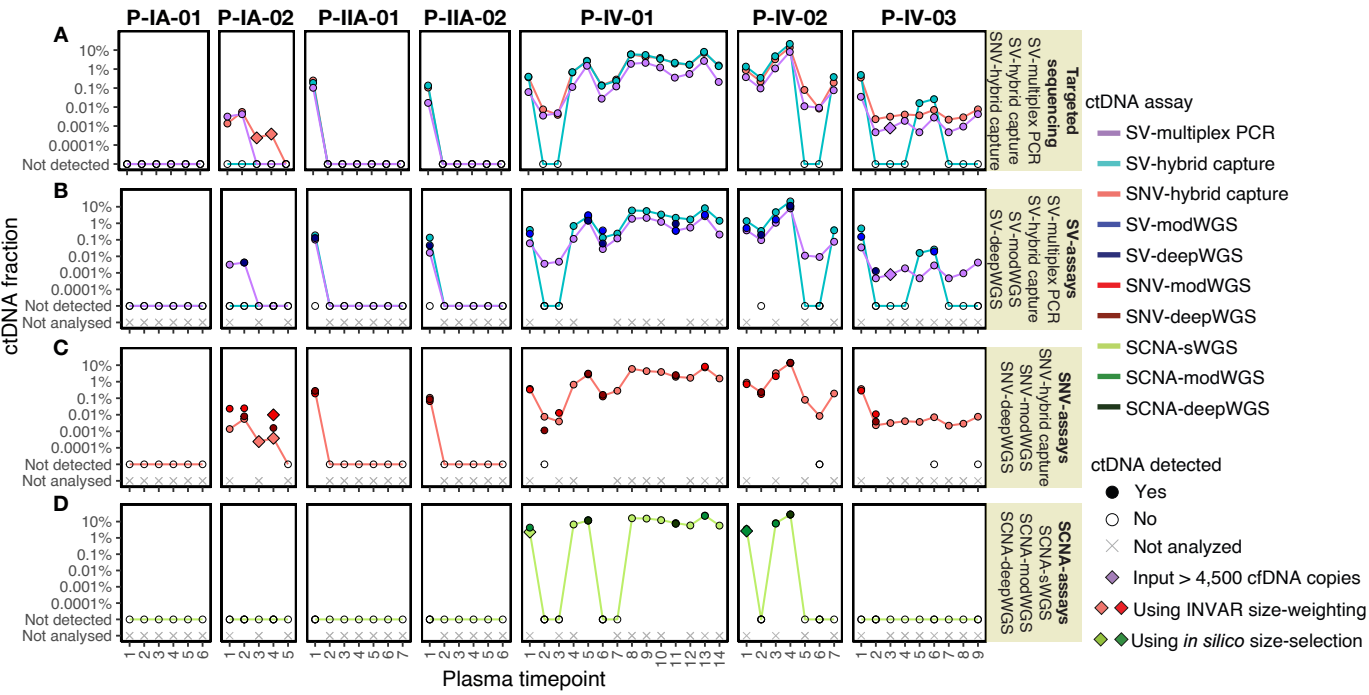

Supplement: EV Figures [file EMS175606-supplement-EV_Figures.zip › Figure EV3.pdf]

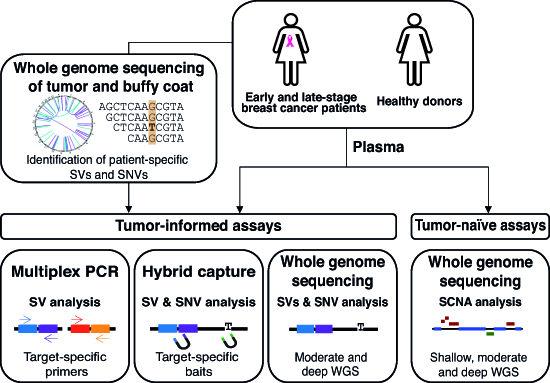

Supplement: Visual Abstract [file EMS175606-supplement-Visual_Abstract.tif]
