# Supplementary material for: Comparison of tumor-informed and tumor-naïve sequencing assays for ctDNA detection in breast cancer
Source: EMBO Mol Med. Author manuscript; Available in PMC 2023 Jun 8. (PMC10245040; doi:10.15252/emmm.202216505)
Supplement: Paper Explained [file EMS175606-supplement-Paper_Explained.docx]

**The paper explained**

Problem:

Circulating tumor DNA (ctDNA) can be used as a non-invasive liquid biopsy in cancer patients to track disease burden in blood. Different strategies have been used to quantify ctDNA, but few studies have compared the performance of different tumor-informed and tumor-naïve assays to detect ctDNA in the same patient samples.

Results:

Our results demonstrate that ctDNA dynamics and tumor allele fractions were highly concordant when targeting different mutation types in serial blood samples collected from breast cancer patients undergoing treatment. Tumour-informed assays showed the highest sensitivity for detection of ctDNA at low concentrations. SNV-hybrid capture, targeting thousands of single nucleotide variants, and SV-multiplex PCR, targeting tens of structural variants, were able to detect ctDNA down to a few parts per million.

Importance:

Choice of assay for ctDNA quantification depends on many factors including the required sensitivity for its intended use, the mutation type being assayed, turnaround time and cost. This study demonstrates that personalized assays targeting patient-specific mutations identified in the tumor were the most sensitive assays to detect low levels of ctDNA in blood, and SV-multiplex PCR has potential to be used a clinical diagnostic assay
