# Supplementary material for: Comparison of tumor-informed and tumor-naïve sequencing assays for ctDNA detection in breast cancer
Source: EMBO Mol Med. Author manuscript; Available in PMC 2023 Jun 8. (PMC10245040; doi:10.15252/emmm.202216505)
Supplement: Synopsis [file EMS175606-supplement-Synopsis.docx]

**Synopsis**

Tumor-informed and tumor-naïve assays were developed to compare detection of circulating tumour DNA in serial plasma samples from patients with stage I-IV breast cancer. These assays targeted structural variants (SVs), single nucleotide variants (SNVs) and somatic copy-number aberrations (SCNAs).

• SNV-hybrid capture, targeting thousands of mutations, was the most sensitive assay, with detection down to an allele fraction (AF) of 0.00024% (2.4 parts per million).

• SV-multiplex PCR analysis of patient-specific rearrangements (targeting as few as 21-47 SVs) also had high sensitivity, with detection down to 0.00047% AF.

•Whole genome sequencing to a mean depth of 20x was able to detect SVs down to 0.02% AF and SNVs to 0.0098% AF

•Deeper WGS to a mean depth of 400x coverage was able to detect SVs down to 0.0013% AF and SNVs to 0.0011% AF.

• Tumor-naïve assays targeting SCNAs were the least sensitive, and increasing the depth of whole genome sequencing (WGS) did not enhance their detection sensitivity.
