# Supplementary material for: Comparison of tumor-informed and tumor-naïve sequencing assays for ctDNA detection in breast cancer
Source: EMBO Mol Med. Author manuscript; Available in PMC 2023 Jun 8. (PMC10245040; doi:10.15252/emmm.202216505)
Supplement: Expanded View Figure Legends [file EV_fig_legs.docx]

Expanded View Figure Legends

**Figure EV1. Circos plots showing patient-specific SVs (structural variants) from all breast cancer patients.** Plots show the number and location of all patient-specific SVs identified by tumor WGS (number in brackets), which were subsequently targeted by the different ctDNA assays (see Table EV2). The breakpoints of all SVs depicted (except those indicated in black) were confirmed in the tumor tissue by multiplex PCR or hybrid capture. Purple lines indicate SVs included in all SV assays (SV-multiplex PCR, SV-hybrid capture, SV-modWGS, and SV-deep- WGS). Turquoise lines indicate SVs analyzed only by SV-hybrid capture, SV-modWGS, and SV-deepWGS but not by SV-multiplex PCR. Black lines indicate 6 SVs identified by WGS of the tumor tissue that were not detected in plasma using any assay (due to no reads observed or homology with repetitive regions) and therefore were removed from further analysis.

**Figure EV2.** **SCNA plots and tumor fraction (TF) observed with the SCNA assays in plasma**

All plasma samples with ctDNA detected either before or after size-selection with SCNA-sWGS, modWGS and deepWGS are plotted as well as tumor and buffy coat from the 7 patients. Plots and tumor fraction (TF) plotted before size-selection for all samples except those marked as “size-selected”; in those samples the plot corresponds to the SCNAs observed after size-selection while the tumor fraction is that generated before size-selection. Tumor fraction estimated from ichorCNA. A-E) SCNA plots from P-IV-01, A) plasma samples detected with SCNA-sWGS, B) plasma samples detected with SCNA-modWGS, C) plasma samples detected with SCNA-deepWGS, D) sWGS of tumor tissue, E) sWGS of buffy coat. F-J) SCNA plots from P-IV-02, F) plasma samples detected with sWGS, G) plasma samples detected with SCNA-modWGS, H) plasma samples detected with SCNA-deepWGS, I) sWGS of tumor tissue, J) sWGS of buffy coat. For both patients, all plasma samples detected have similar alterations between different plasma samples from the same patient tested with the different SCNA assays as well as with the tumor tissue. K-T) sWGS of tumor and buffy coat from the remaining 5 patients with no plasma sample detected by the analysis of SCNAs.

**Figure EV3. ctDNA detection and fractions using the different ctDNA assays.** ctDNA fractions are plotted as an allele fraction for SV/SNV assays and as tumor fraction for SCNA assays. Plots (A-D) show comparisons of the detection and ctDNA fraction obtained with different assays. A) Comparison of targeted-sequencing assays (SV-multiplex PCR, SV-hybrid capture, SNV-hybrid capture); B) Comparison of all assays targeting SVs (SV-multiplex PCR, SV-hybrid capture, SV-modWGS, SV-deepWGS); C) Comparison of all assays targeting SNVs (SNV-hybrid capture, SNV-modWGS, SNV-deepWGS); D) Comparison of all assays evaluating SCNAs (SCNA-sWGS, SCNA-modWGS, SCNA-deepWGS). The individual plots and number of samples assayed are shown in Figure 3.

**Figure EV4. Clinical information and detection of ctDNA in each patient using targeted ctDNA assays.**

The shaded boxes represent different treatment periods and the vertical dotted lines mark the time of diagnosis (black), time of surgery where appropriate (dark orange) and the time of tissue collection for whole genome sequencing (dark green). Days indicated on the x axis refer to the number of days before or after the collection of the first plasma sample. Two samples were taken at day 0 (D0) in stage IA patients (P-IA-01, P-IA-02), before (T1) and after surgery (T2). Individual plots showing the allele fraction from every ctDNA assay can be found in Figure 3. *RT: radiotherapy; FEC: 5 fluorouracil (5FU), epirubicin and cyclophosphamide; EC: epirubicin and cyclophosphamide; /T followed by docetaxel; ND: Not detected.*
