# Supplementary material for: Comparison of tumor‐informed and tumor‐naïve sequencing assays for ctDNA detection in breast cancer
Source: EMBO Mol Med. 2023 May 10;15(6):e16505. doi: 10.15252/emmm.202216505 (PMC10245040; doi:10.15252/emmm.202216505)
Supplement: Supplementary file 3 [file EMMM-15-e16505-s012.pdf]

EMBO Molecular Medicine

Comparison of tumor-informed and tumor-naive sequencing assays for ctDNA detection in breast cancer

Davina Gale, Angela Santonja, Wendy Cooper, Matthew Eldridge, Paul Edwards, James Morris, Abigail Edwards, Hui Zhao, Katrin Heider, Dominique-Laurent Couturier, Aadhitthya Vijayaraghavan, Paulius Mennea, Chris Smith, Chris Boursnell, Raquel García, Oscar Rueda, Emma Beddowes, Heather Biggs, Stephen Sammut, Nitzan Rosenfeld, Carlos Caldas, Jean Abraham, and Emma-Jane Ditter
DOI: 10.15252/emmm.202216505

Corresponding author(s): Davina Gale (davina.gale@cruk.cam.ac.uk) , Jean Abraham (ja344@medschl.cam.ac.uk)

Review Timeline:

Submission Date:	28th Jun 22
Editorial Decision:	30th Jun 22
Appeal Received:	4th Jul 22
Editorial Decision:	16th Aug 22
Revision Received:	8th Nov 22
Editorial Decision:	24th Nov 22
Revision Received:	17th Feb 23
Editorial Decision:	2nd Mar 23
Revision Received:	5th Apr 23
Accepted:	6th Apr 23

Editor: Lise Roth

Transaction Report:

(Note: With the exception of the correction of typographical or spelling errors that could be a source of ambiguity, letters and reports are not edited. Depending on transfer agreements, referee reports obtained elsewhere may or may not be included in this compilation. Referee reports are anonymous unless the Referee chooses to sign their reports.)

30th Jun 2022

Decision on your manuscript EMM-2022-16505

Dear Dr. Gale,

Thank you for submitting your manuscript to EMBO Molecular Medicine. I have now read your article carefully and discussed it with the other members of our editorial team. I am sorry to inform you that we find that the manuscript is not well suited for publication in EMM, and that we therefore have decided not to proceed with peer review. However, our colleagues from our sister journal Life Science Alliance would be happy to offer peer review if you were to transfer your manuscript there. Life Science Alliance is an open access journal launched in partnership between EMBO Press, Rockefeller Press, and Cold Spring Harbor Laboratory Press, and publishes work that is of high value to the respective communities across all areas in the life sciences.

We appreciate that your study reports the development and analysis of different tumor-informed and tumor-naïve assays for the quantification of circulating tumor DNA (ctDNA) using the same samples from 7 patients with different stages of breast cancer, and presents a thorough comparison of these methods. However, due to the limited number of patients with different clinical characteristics, we feel your study is rather specialized for the broad audience of EMBO Molecular Medicine, and I am therefore afraid we cannot offer further consideration here.

That being said - and as mentioned above - my colleagues would be pleased to send your manuscript out for formal peer-review at Life Science Alliance in its current form. You can take advantage of this offer by transferring your study to Life Science Alliance using the link below; no re-formatting is required.

I am very sorry to disappoint you on this occasion and would like to reassure you that this is not a judgment of the quality or interest of your work, but a decision based on the scope requirement of our journal.

With kind regards,

Lise Roth

Lise Roth, PhD
Senior Editor
EMBO Molecular Medicine

=========================

As a service to authors, EMBO provides authors with the possibility to transfer a manuscript that one journal cannot offer to publish to another EMBO publication. The full manuscript and if applicable, reviewers reports are automatically sent to the receiving journal to allow for fast handling and a prompt decision on your manuscript. For more details of this service, and to transfer your manuscript to another EMBO title please click on Link Not Available

Please do not share this URL as it will give anyone who clicks it access to your account.

Dear Dr Roth,

EMM-2022-16505: 'Comparison of tumor-informed and tumor-naive sequencing assays for ctDNA detection in breast cancer'

Thank you for your email. We appreciate the time taken to review our manuscript, and many thanks for your helpful feedback.

Naturally we are disappointed that, on consideration, this will not be sent for peer review. We understand that this is data from 7 breast cancer patients, but we believe this study is by far the most comprehensive analysis comparing the performance of analysing different genomic alterations (SNVs, SVs and SCNAs) using multiple cutting-edge methods in the same patient cohort, and evaluating the performance of both tumor-informed and tumor-naive assays.

We have been able to monitor longitudinal ctDNA dynamics across 54 samples from stage 1A - IV breast cancer patients and 19 samples from healthy donors using up to 10 different assays, including multiplex PCR (SV-multiplex PCR), hybrid capture (SNV-hybrid capture, SV-hybrid capture) and different depths of whole genome sequencing (SV-modWGS, SV-deepWGS, SNV-deepWGS) in addition to using a tumor-naïve approach to analyse copy-number aberrations (SCNA-sWGS, SCNA-modWGS and SCNA-deepWGS). Given the current costs of sequencing, it would be cost prohibitive to provide such a comprehensive performance evaluation in a wider clinical cohort, and we aimed to mitigate these concerns by analysing longitudinal data from stage 1A to stage IV patients undergoing treatment, thereby evaluating assay performance across the full range of ctDNA levels expected in a larger cohort.

This provides a unique dataset for the scientific community, providing a comprehensive assessment of the performance of different mutation types using both personalized and non-personalized approaches, demonstrating the assay with highest sensitivity was SNV-hybrid capture, with ctDNA detection down to an allele fraction of 0.00024% (2.4 parts per million). We also demonstrate that the SV multiplex PCR assay was able to detect ctDNA down to 0.00047% AF (4.7 parts per million), and has potential as a clinical assay for MRD detection. Furthermore, the deepWGS data provides useful and timely insight into what may be feasible in the future as sequencing costs decrease, and provides comparative data which many researchers would not have the resources to be able to access at this time.

Given these considerations, we hope that on reflection you may reconsider the decision and sent the manuscript for peer review.

We look forward to hearing from you.

Best regards,

Dr Davina Gale

On behalf of the study team

2nd Editorial Decision 16th Aug 2022

16th Aug 2022

Dear Dr. Gale,

Thank you for the submission of your manuscript to EMBO Molecular Medicine.
As mentioned in a previous correspondence, following your appeal, we consulted with an external expert in the field and decided to send your manuscript out for review.
We have now received feedback from two of the three reviewers who agreed to evaluate your manuscript. Referee #1 had unexpected circumstances and has not yet been able to provide his/her report. Given that both referees #2 and #3 provide similar recommendations, we prefer to make a decision now in order to avoid further delay in the process. Should referee #1 provide a report, we will send it to you, with the understanding that we will not ask you extensive experiments in addition to the ones required in the enclosed reports from referees #2 and #3.

As you will see from the reports below, the referees acknowledge the interest of the study and are overall supporting publication of your work pending appropriate revisions. Addressing the reviewers' concerns in full will be necessary for further considering the manuscript in our journal, and acceptance of the manuscript will entail a second round of review. EMBO Molecular Medicine encourages a single round of revision only and therefore, acceptance or rejection of the manuscript will depend on the completeness of your responses included in the next, final version of the manuscript. For this reason, and to save you from any frustrations in the end, I would strongly advise against returning an incomplete revision.

Revised manuscripts should be submitted within three months of a request for revision; they will otherwise be treated as new submissions, except under exceptional circumstances in which a short extension is obtained from the editor.

When submitting your revised manuscript, please carefully review the instructions that follow below. We perform an initial quality control of all revised manuscripts before re-review; failure to include requested items will delay the evaluation of your revision.

We require:

1) A .docx formatted version of the manuscript text (including legends for main figures, EV figures and tables). Please make sure that the changes are highlighted to be clearly visible.

2) Individual production quality figure files as .eps, .tif, .jpg (one file per figure). For guidance, download the 'Figure Guide PDF' (https://www.embopress.org/page/journal/17574684/authorguide#figureformat).

3) A .docx formatted letter INCLUDING the reviewers' reports and your detailed point-by-point responses to their comments. As part of the EMBO Press transparent editorial process, the point-by-point response is part of the Review Process File (RPF), which will be published alongside your paper.

4) A complete author checklist, which you can download from our author guidelines (https://www.embopress.org/page/journal/17574684/authorguide#submissionofrevisions). Please insert information in the checklist that is also reflected in the manuscript. The completed author checklist will also be part of the RPF.

5) Please note that all corresponding authors are required to supply an ORCID ID for their name upon submission of a revised manuscript (currently missing for Jean Abraham).

6) It is mandatory to include a 'Data Availability' section after the Materials and Methods. Before submitting your revision, primary datasets produced in this study need to be deposited in an appropriate public database, and the accession numbers and database listed under 'Data Availability'. Please remember to provide a reviewer password if the datasets are not yet public (see https://www.embopress.org/page/journal/17574684/authorguide#dataavailability).
In case you have no data that requires deposition in a public database, please state so in this section. Note that the Data Availability Section is restricted to new primary data that are part of this study.

7) For data quantification: please specify the name of the statistical test used to generate error bars and P values, the number (n) of independent experiments (specify technical or biological replicates) underlying each data point and the test used to calculate p-values in each figure legend. The figure legends should contain a basic description of n, P and the test applied. Graphs must include a description of the bars and the error bars (s.d., s.e.m.). Please provide exact p values.

8) We would also encourage you to include the source data for figure panels that show essential data. Numerical data should be provided as individual .xls or .csv files (including a tab describing the data). For blots or microscopy, uncropped images should be submitted (using a zip archive if multiple images need to be supplied for one panel). Additional information on source data and instruction on how to label the files are available at
.

9) Our journal encourages inclusion of *data citations in the reference list* to directly cite datasets that were re-used and obtained from public databases. Data citations in the article text are distinct from normal bibliographical citations and should directly link to the database records from which the data can be accessed. In the main text, data citations are formatted as follows: "Data ref: Smith et al, 2001" or "Data ref: NCBI Sequence Read Archive PRJNA342805, 2017". In the Reference list, data citations must be labeled with "[DATASET]". A data reference must provide the database name, accession number/identifiers and a resolvable link to the landing page from which the data can be accessed at the end of the reference. Further instructions are available at .

10) We replaced Supplementary Information with Expanded View (EV) Figures and Tables that are collapsible/expandable online. A maximum of 5 EV Figures can be typeset. EV Figures should be cited as 'Figure EV1, Figure EV2" etc... in the text and their respective legends should be included in the main text after the legends of regular figures.
- For the figures that you do NOT wish to display as Expanded View figures, they should be bundled together with their legends in a single PDF file called *Appendix*, which should start with a short Table of Content. Appendix figures should be referred to in the main text as: "Appendix Figure S1, Appendix Figure S2" etc.
- Additional Tables/Datasets should be labeled and referred to as Table EV1, Dataset EV1, etc. Legends have to be provided in a separate tab in case of .xls files. Alternatively, the legend can be supplied as a separate text file (README) and zipped together with the Table/Dataset file.
See detailed instructions here:
.

11) The paper explained: EMBO Molecular Medicine articles are accompanied by a summary of the articles to emphasize the major findings in the paper and their medical implications for the non-specialist reader. Please provide a draft summary of your article highlighting
- the medical issue you are addressing,
- the results obtained and
- their clinical impact.
This may be edited to ensure that readers understand the significance and context of the research. Please refer to any of our published articles for an example.

12) For more information: There is space at the end of each article to list relevant web links for further consultation by our readers. Could you identify some relevant ones and provide such information as well? Some examples are patient associations, relevant databases, OMIM/proteins/genes links, author's websites, etc...

13) Every published paper now includes a 'Synopsis' to further enhance discoverability. Synopses are displayed on the journal webpage and are freely accessible to all readers. They include a short stand first (maximum of 300 characters, including space) as well as 2-5 one-sentences bullet points that summarizes the paper. Please write the bullet points to summarize the key NEW findings. They should be designed to be complementary to the abstract - i.e. not repeat the same text. We encourage inclusion of key acronyms and quantitative information (maximum of 30 words / bullet point). Please use the passive voice. Please attach these in a separate file or send them by email, we will incorporate them accordingly.

Please also suggest a striking image or visual abstract to illustrate your article as a PNG file 550 px wide x 300-600 px high.

14) As part of the EMBO Publications transparent editorial process initiative (see our Editorial at http://embomolmed.embopress.org/content/2/9/329), EMBO Molecular Medicine will publish online a Review Process File (RPF) to accompany accepted manuscripts.
In the event of acceptance, this file will be published in conjunction with your paper and will include the anonymous referee reports, your point-by-point response and all pertinent correspondence relating to the manuscript. Let us know whether you agree with the publication of the RPF and as here, if you want to remove or not any figures from it prior to publication.
Please note that the Authors checklist will be published at the end of the RPF.

EMBO Molecular Medicine has a "scooping protection" policy, whereby similar findings that are published by others during review or revision are not a criterion for rejection. Should you decide to submit a revised version, I do ask that you get in touch after three months if you have not completed it, to update us on the status.

I look forward to receiving your revised manuscript.

Yours sincerely,

Lise Roth

Lise Roth, PhD

Senior Editor
EMBO Molecular Medicine

***** Reviewer's comments *****

Referee #2 (Comments on Novelty/Model System for Author):

The technical quality of this study needs to be improve showing/assaying the corresponding controls for all the assays showed in the article.
The novelty is high since there are no studies comparing this number of technologies/methodologies for ctDNA detection in cancer.
However, the medical impact is substantially impacted by the number of patients included in the study.

Referee #2 (Remarks for Author):

In this study, Santonja A, Cooper NW and colleagues present a study in which they performed an important number of technologies and methodologies, using different types of tumour aberrations to detect ctDNA in localized and metastatic breast cancer (BC). Importantly, these approaches are both informed and non-informed from tumour tissue sequencing, this last of a great interest.
However, the study is hardly impacted by the number of patients included as the authors clearly specify in the discussion section despite a quite interesting number of plasma samples have been assayed.

Major comments:

1. In this type of studies, where ctDNA identification moves in the technologies´ limits of detection, assaying the appropriate negative controls are paramount. In general, I did not see any information about the controls for each of the technologies applied. These assays should be accompanied by both germline controls from the same patient, ideally from each timepoint, and plasma controls from healthy individuals. The authors stated that healthy controls were included for some of the assays but it is not clear where, how many and the sequencing stats of these controls.
If not for all timepoints, germline controls should be assayed for all assays as well as plasma controls from unrelated healthy individuals. If they have been already assayed, as the authors specify in some paragraphs in the results section, sequencing stats should be shown in a Supplementary Table. Sequencing of control samples should mirror the sequencing performed from patient´s samples.

2. In the dilution experiments, did the authors included the convenient controls using the matched patient´s germline DNA and cfDNA from unrelated healthy individuals? If yes, sequencing stats should be shown in a Supplementary Table. If not, this should be included.

3. For the SCNAs detection, the authors should include which CNAs were detected in the plasma samples and the status both in tumour tissue and in the corresponding germline DNA including plasma from healthy individuals, in a Supplementary Table. Using another technology for this purpose would be interesting, for example ddPCR.

4. Overall, discussion should be reduced since is repeating much of the information reported in the results section. In addition, the discussion should make clear that it is not possible to conclude that these assays have a utility to detect MRD since there are not enough patients/relapses after surgery in the early BC setting to conclude anything in this respect.

Minor comments:

1. Figure 2 is very limited in terms of information for the reader. This should be move to supplementary material.

2. Figure 4. The comparison between assays should be moved to and independent figure and to supplementary materials since is repeating information.

3. The Supplementary Table 4 is confusing regarding the number of libraries assayed, specially from the "Multiplex PCR" row. This should be clarified in the legend.

4. Figure 5 representing different SCNAs analyses in two metastatic patients should be also move to supplementary materials.

5. The authors should clarify in the figures where the timepoints are represented, which timepoints have been assayed in terms of timing before and after surgery for the early-stage BC and timing after diagnosis in the metastatic ones instead of numbers.

Referee #3 (Comments on Novelty/Model System for Author):

It is a very interesting study with low data in the literature exposing clearly the results of different method of ctDNA detection and the methods used is of interest. Clearly there is no novelty but one again the results are interesting. The medical impact is indirect but it could participate to clarify the issues of the different methods of ctDNA detection

Referee #3 (Remarks for Author):

This article addresses a very interesting question regarding the different approaches to demonstrate cDNA in the plasma of cancer patients. The objective of this article is to compare two approaches: one agnostic of tumor genetic alterations, the other not. These comparisons are also done with different sequencing methods (multiplex PCR, hybrid capture and different whole genome sequencing with different depths). This comparison is performed on 7 breast cancer patients, 4 early stages and 3 advanced stages. Several plasma samples were collected during the course of the disease and 54 samples were analyzed in the comparison. The results are clearly presented, and the comparison of the different methods is very well documented; the figures illustrate the results appropriately.
I have a minor concern in the discussion section the authors more clearly the cost of the different methods (a table could be useful) and a strategy could be presented to detect ctDNA in the two scenario cases early and late stage.

Author’s Point-by-Point response to Reviewer’s comments
We thank the Reviewers for their helpful comments, which we agree has significantly enhanced our manuscript. Below we include a detailed point-by-point response to each of the Reviewers’ comments.

******* Reviewer's comments *******

Referee #2 (Comments on Novelty/Model System for Author):

The technical quality of this study needs to be improve showing/assaying the corresponding controls for all the assays showed in the article.
The novelty is high since there are no studies comparing this number of technologies/methodologies for ctDNA detection in cancer.
However, the medical impact is substantially impacted by the number of patients included in the study.

Referee #2 (Remarks for Author):

In this study, Santonja A, Cooper NW and colleagues present a study in which they performed an important number of technologies and methodologies, using different types of tumour aberrations to detect ctDNA in localized and metastatic breast cancer (BC). Importantly, these approaches are both informed and non-informed from tumour tissue sequencing, this last of a great interest. However, the study is hardly impacted by the number of patients included as the authors clearly specify in the discussion section despite a quite interesting number of plasma samples have been assayed.

We thank Reviewer #2 for their comments, and for recognising the novelty of this study . We appreciate that this is data from just 7 breast cancer patients, but believe this study is the most comprehensive analysis to date comparing the performance of different tumor-informed and tumor-naïve ctDNA assays targeting different genomic alterations (SNVs, SVs and SCNAs) using multiple cutting-edge methods in the same patient cohort. We have been able to monitor longitudinal ctDNA dynamics across 54 plasma samples from stage 1A - IV breast cancer patients and 19 samples from healthy donors using up to 10 different assays, including multiplex PCR (SV-multiplex PCR), hybrid capture (SNV-hybrid capture, SV-hybrid capture) and different depths of whole genome sequencing

(SV-modWGS, SV-deepWGS, SNV-deepWGS). In addition we used a tumor-naïve approach to analyse copy-number aberrations (SCNA-sWGS, SCNA-modWGS and SCNA-deepWGS).

Given the current costs of sequencing, it would be cost prohibitive to provide such a comprehensive performance evaluation in a wider clinical cohort. We aimed to mitigate these concerns by analysing longitudinal data from stage IA to stage IV patients undergoing treatment, thereby evaluating assay performance across a wide range of ctDNA levels expected in a larger cohort. The deepWGS data (~400x mean sequencing coverage) provides a useful and timely insight into what may be feasible in the future as sequencing costs decrease, and provides comparative data which many researchers will not have the resources to be able to access at this time.

Major comments:

1. In this type of studies, where ctDNA identification moves in the technologies´ limits of detection, assaying the appropriate negative controls are paramount. In general, I did not see any information about the controls for each of the technologies applied. These assays should be accompanied by both germline controls from the same patient, ideally from each timepoint, and plasma controls from healthy individuals. The authors stated that healthy controls were included for some of the assays but it is not clear where, how many and the sequencing stats of these controls.
If not for all timepoints, germline controls should be assayed for all assays as well as plasma controls from unrelated healthy individuals. If they have been already assayed, as the authors specify in some paragraphs in the results section, sequencing stats should be shown in a Supplementary Table. Sequencing of control samples should mirror the sequencing performed from patient´s samples.

We thank Reviewer #2 for their comments. We would like to note that information on the germline and healthy donor plasma control samples used to assess performance of all assays was detailed in the original manuscript in Materials and Methods (page 33 – 50 in the revised '**EMM-2022-16505-V2-Q_Manuscript_tracked.docx'** document), and is now clearly stated in Table EV6 and in Results. Given Reviewer #2's concerns, and to help provide further clarification to the reader, we have now included detailed information in every appropriate section of Results regarding the number of samples and controls analyzed in each assay and included results on negative controls. We have also stated in every appropriate section the results on tumor and buffy coat analysis and included plots showing somatic copy-number analysis of tumor and buffy coat samples (Figure 4 and Figure EV2). To clarify the sequencing statistics, Table EV6 has been updated to separately present the stats on plasma samples from patients and healthy donors, as well as tumor and buffy coat.

As noted in both Materials and Methods and Results, for the development of all tumor-informed assays (SV-multiplex PCR, SV-hybrid capture, SNV-hybrid capture, SNV-modWGS and SNV-deepWGS), tumor-specific mutations were first identified by whole genome sequencing of both tumor and matched buffy coat sample from each patient, to ensure that SVs and SNVs targeted in downstream assays were unique to the tumor, and not present in matched germline samples. Please note that multiple buffy coat samples were not available for analysis.

In the development of the SV-multiplex PCR assays, tumor-specific SVs were first identified by sequencing of tumor and germline DNA from each patient. Next primers were designed to target sequences either side of the predicted SV breakpoints, and then tested on both matched tumor and buffy coat DNA from each patient to ensure that only tumor-specific variants were assayed in the final multiplex pools, as detailed in Materials and Methods (page 39 – 40, line 971 – 977). SV-multiplex PCR assays were used to assess ctDNA levels in longitudinal plasma samples from breast cancer patients, and also using plasma from a pool of 5 healthy donors as negative controls and water as a no-template control (page 42, line 1024 – 1028).

In the SV- and SNV-hybrid capture assays, mutations were first identified by whole genome sequencing of both tumor and germline samples from each patient as detailed above. Sequencing libraries were prepared from fragmented tumor DNA from the 7 breast cancer patients, matched fragmented buffy coat DNA, and plasma cell-free DNA from 54 patient plasma samples and 18 individual healthy donor plasma samples. Using hybrid capture, ctDNA was not detected in any of the healthy plasma samples by SV analysis, or for SNVs analysed by INVAR where plasma from healthy controls was used to assess specificity. Given that the targeted variants are patient-specific, we were able to also leverage other patients' samples as controls to assess background noise, as previously described (Wan et al., Science Translational Medicine, 2020, and referred to in the Statistical considerations section, page 35 - 36, line 879-885). The use of data from plasma DNA samples from other patients is a cost-effective way to access sequencing data from loci of interest and assess background noise rates.

For the tumor-naïve assays targeting SCNAs, we performed shallow, moderate and deep whole genome sequencing. In the sWGS assays, sequencing was performed on 54 patient plasma samples and plasma from 18 healthy donors to a mean sequencing depth of 1.2x. As requested, we have now included data on both tumor and germline SCNAs identified by shallow WGS in the manuscript (detailed in Results page 17, 401 – 446, and shown in Figure 4 and Figure EV2). We thank the Reviewer as this helps to make it clear that the SCNAs identified in patient plasma have a similar profile to those identified in the tumor but not germline DNA, and are tumor-specific aberrations not present in the germline.

As detailed in Methods, and now more clearly stated in Results (page 19, line 450 – 451), we performed moderate WGS on 21 plasma samples from breast cancer patients and 4 from individual healthy donors to a mean sequencing depth of ~20x. Given the costs of deep sequencing (mean coverage of ~400x), costs prohibited us from analysing more than 12 samples, which was performed on samples from breast cancer patient samples with a wide range of expected AFs (0.0003%-13.8% AF) to help assess the performance of the assay in samples with different levels of tumor burden.

2. In the dilution experiments, did the authors included the convenient controls using the matched patient´s germline DNA and cfDNA from unrelated healthy individuals? If yes, sequencing stats should be shown in a Supplementary Table. If not, this should be included.

In the dilution experiments to assess the performance of the patient-specific SV-multiplex PCR assays, a tumor DNA dilution series experiment was performed for each patient-specific assay, testing a range of tumor DNA dilutions between 10% AF – 0.0004% AF, and using matched buffy coat and water as negative controls. In addition to being included in the Methods, we have now added this information to the Results section (page 9, line 203 – 215) to help make this clearer to the reader. Sequencing stats are shown in Table EV6. All SV-multiplex assays (i.e. all primer pools) were also tested in cfDNA from unrelated healthy individuals in addition to being assayed in plasma samples from patients.

3. For the SCNAs detection, the authors should include which CNAs were detected in the plasma samples and the status both in tumour tissue and in the corresponding germline DNA including plasma from healthy individuals, in a Supplementary Table. Using another technology for this purpose would be interesting, for example ddPCR.

We thank Reviewer #2 for this suggestion. As requested, we have now included data on both tumor and germline SCNAs identified by shallow WGS in the manuscript (Results page 18, line 440 - 443;

page 19, line 453 – 455; Figure 4 and Figure EV2). The SCNAs observed in plasma from stage IV patients show similar profiles to those seen in analysis of tumour samples from the same patients, but were not observed when analysing matched buffy coat samples. This confirms that the SCNAs identified are tumor-specific aberrations not present in the germline. For those earlier stage patients where SCNAs were not observed in plasma, Figure EV2 clearly shows that copy number abberrations were present in the tumor. They were likely not detected in plasma due lower fractions below the limit of detection of the assay. To clarify the results on healthy indiviuduals, we have included a statement on pages 17 and 18 (lines 416 – 418 and lines 445 – 446). Sequencing statistics on healthy controls and buffy coat are now shown in Table EV6.

4. Overall, discussion should be reduced since is repeating much of the information reported in the results section. In addition, the discussion should make clear that it is not possible to conclude that these assays have a utility to detect MRD since there are not enough patients/relapses after surgery in the early BC setting to conclude anything in this respect.

Thank you for these comments. We have now reduced the length of the Discussion and omitted the conclusion section to avoid repeating information. We have also removed any unintended implication that the assays were able to be used to detect MRD given our limited sample size.

Minor comments:

1.Figure 2 is very limited in terms of information for the reader. This should be move to supplementary material.

Thank you for this suggestion. We have moved Figure 2 to Supplementary Figure EV1.

2. Figure 4. The comparison between assays should be moved to and independent figure and to supplementary materials since is repeating information.

Thank you for this suggestion. As requested, we have moved Figure 4 k-n comparisons to Figure EV3. However, we do believe that the comparison between assays is an important part of the paper to directly compare ctDNA dynamics and observed allele fractions using different assays, so ask the editor to consider whether this should remain part of the main figure or be in EV format.

3. The Supplementary Table 4 is confusing regarding the number of libraries assayed, specially from the "Multiplex PCR" row. This should be clarified in the legend.

Thank you for your comments. We have now updated Table EV6 (formerly Supplementary Table 4) to describe the statistics per sample assayed rather than per library assayed to help make it clearer. We have also separated the sequencing reads from plasma samples and from healthy donors as well as from tumor and buffy coat in order show that similar number of reads were obtained for samples and controls. We have made further clarifications in the legend of this table.

4. Figure 5 representing different SCNAs analyses in two metastatic patients should be also move to supplementary materials.

Thank you for your comments. As requested above, we have now included data in Figure 4 (which was Figure 5 in the previously submitted version) showing tumor and germline sWGS data. This makes it clearer to the reader that the SCNAs identified in patient plasma have a similar profile to those identified in the tumor but were not present in germline DNA. Given this, we have left the

figure where it is currently, but would be happy to move it to EV if required. Please advise where you would like this figure to be incorporated.

5. The authors should clarify in the figures where the timepoints are represented, which timepoints have been assayed in terms of timing before and after surgery for the early-stage BC and timing after diagnosis in the metastatic ones instead of numbers.

To help clarify, Figure EV4 (formerly Supplementary Figure 2) has been updated to show the days from diagnosis to plasma timepoint 1. Figure EV4 and Table EV1 also depict the timepoint number and days elapsed before and after surgery in both early and late stage patients We have also clarified the relationship between each timepoint analyzed and the number of days after plasma timepoint 1 in Figure EV4 and Table EV9.

Referee #3 (Comments on Novelty/Model System for Author):

It is a very interesting study with low data in the literature exposing clearly the results of different method of ctDNA detection and the methods used is of interest. Clearly there is no novelty but one again the results are interesting. The medical impact is indirect but it could participate to clarify the issues of the different methods of ctDNA detection

Referee #3 (Remarks for Author):

This article addresses a very interesting question regarding the different approaches to demonstrate cDNA in the plasma of cancer patients. The objective of this article is to compare two approaches: one agnostic of tumor genetic alterations, the other not. These comparisons are also done with different sequencing methods (multiplex PCR, hybrid capture and different whole genome sequencing with different depths). This comparison is performed on 7 breast cancer patients, 4 early stages and 3 advanced stages. Several plasma samples were collected during the course of the disease and 54 samples were analyzed in the comparison. The results are clearly presented, and the comparison of the different methods is very well documented; the figures illustrate the results appropriately.
I have a minor concern in the discussion section the authors more clearly the cost of the different methods (a table could be useful) and a strategy could be presented to detect ctDNA in the two scenario cases early and late stage.

We thank Reviewer #3 for their encouraging comments and interest in our study. Regarding the discussion, we have now included a strategy of when specific assays could ideally be used in different scenarios. The tumor-naïve sWGS assays are most appropriately used to assess ctDNA in patients with late stage disease, given we have demonstrated that these assays can detect SCNAs in patients with stage IV breast cancer (but not in patients with early-stage disease) and can be performed at relatively low cost with a rapid turnaround time. In contrast, the tumor-informed assays have sensitivity for detection of low levels of ctDNA down to a few parts per million, and are most appropriate for use in patients with low burden disease where assay sensitivity is of critical importance.

Given the reviewers concern on lack of clarity regarding costs, we have decided to remove any reference to specific costs, given that these can be highly variable depending on country-specific and lab-specific discount pricing structures, and that fluctuations that are likely to occur over time. We have kept in that a $100 genome may become a reality in the future, given that the recent press releases from both Ultima Genomics and Illumina indicate that recent sequencing technology innovations are enabling an advancement towards this goal.

24th Nov 2022

Dear Dr. Gale,

Thank you for the submission of your manuscript to EMBO Molecular Medicine. We have now received feedback from the two referees who originally reviewed your manuscript. As you will see below, while referees #3 is supportive of publication, referee #2 still has a concern related to the absence of sequencing data from healthy individuals.
We would therefore like to invite you to further revise your manuscript to address this remaining concern.

Moreover, please also address the following editorial points:
- Acknowledgments: Please make sure that the funding sources mentioned in the manuscript match those entered in the submission system.
- For each EV table, please add the legend in a separate tab.
- Please make sure that all figures and figure panels are referenced in the manuscript text. Figure callouts are currently missing for Fig. 3E and Fig. 4E-G. Moreover, Fig. 3H-J should be called out before Fig. 4.
- I slightly edited your synopsis text to fit our style and format, please let me know if you agree with the following or amend as you see fit:

Tumor-informed and tumor-naïve assays were developed to compare detection of circulating tumour DNA in serial plasma samples from patients with stage I-IV breast cancer. These assays targeted structural variants (SVs), single nucleotide variants (SNVs) and somatic copy-number aberrations (SCNAs).
• SNV-hybrid capture, targeting thousands of mutations, was the most sensitive assay, with detection down to an allele fraction (AF) of 0.00024% (2.4 parts per million).
• SV-multiplex PCR analysis of patient-specific rearrangements (targeting as few as 21-47 SVs) also had high sensitivity, with detection down to 0.00047% AF.
•Whole genome sequencing to a mean depth of 20x was able to detect SVs down to 0.02% AF and SNVs to 0.0016% AF
•Deeper WGS to a mean depth of 400x coverage was able to detect SVs down to 0.0013% AF and SNVs to 0.0011% AF.
• Tumor-naïve assays targeting SCNAs were the least sensitive, and increasing the depth of whole genome sequencing (WGS) did not enhance their detection sensitivity.

Thank you for providing a nice synopsis picture. Please resize it as a PNG/TIFF/JPEG file 550 px wide x 300-600 px high. The text should remain legible.

We note that you agree with the publication of the Review Process File, that will include the anonymous referee reports, your point-by-point response and all pertinent correspondence relating to the manuscript. Please note that the Authors checklist will be published at the end of the RPF.

I look forward to receiving your revised manuscript.

With kind regards,

Lise Roth

Lise Roth, PhD
Senior Editor
EMBO Molecular Medicine

***** Reviewer's comments *****

Referee #2 (Comments on Novelty/Model System for Author):

The technical quality of the study is potentially high but I still have concerns about the absence of the sequencing data from

the

healthy individuals plasma.
The novelty is high since there are no studies comparing these type of sequencing-intensive assays.
However, the medical impact is low given the low number of patients included.

Referee #2 (Remarks for Author):

In this revised version of the manuscript, the authors make clear that the buffy coat was used to select tumour-specific variants in the assays. This was clear to me in the first version but now they are also showing some results in this regard.
Showing complete sequencing data from healthy individual plasma is paramount in these type of studies to control the sequencing noise. It is not enough by stating that ctDNA detection was negative in healthy plasma controls in the text. For example, I have concerns that when analysing 1,347 - 7,491 SNVs there is absolutetly no sequencing noise in the plasma from healthy controls. cfDNA control should be assayed in every determination for everytime point to control for noise especially for SNVs and this is not clear to me.

On the other hand, I suggest that this study could be a short report given the included number of patients.

Referee #3 (Comments on Novelty/Model System for Author):

The authors responded adequately to my remarks. It is an interesting work that deserves to be published even if it is not new and with a medical impact that can be qualified as medium

Author's Point-by-Point response to Reviewers' comments

***** Reviewer's comments *****

Referee #2 (Comments on Novelty/Model System for Author):

The technical quality of the study is potentially high but I still have concerns about the absence of the sequencing data from the healthy individuals plasma.
The novelty is high since there are no studies comparing these type of sequencing-intensive assays. However, the medical impact is low given the low number of patients included.

Referee #2 (Remarks for Author):

In this revised version of the manuscript, the authors make clear that the buffy coat was used to select tumour-specific variants in the assays. This was clear to me in the first version but now they are also showing some results in this regard.
Showing complete sequencing data from healthy individual plasma is paramount in these type of studies to control the sequencing noise. It is not enough by stating that ctDNA detection was negative in healthy plasma controls in the text. For example, I have concerns that when analysing 1,347 - 7,491 SNVs there is absolutetly no sequencing noise in the plasma from healthy controls. cfDNA control should be assayed in every determination for everytime point to control for noise especially for SNVs and this is not clear to me.

On the other hand, I suggest that this study could be a short report given the included number of patients.

We would like to thank Referee #2 for their comments, and apologise that information on sequencing noise in healthy control plasma was not sufficiently clear in the manuscript.

For SNV-hybrid capture, SNV-modWGS, SNV-deepWGS assays using INVAR analysis

To help further clarify, for INVAR analysis of thousands of SNVs using SNV-hybrid capture, SNV-modWGS and SNV-deepWGS, we have now included Table EV12, which shows the number of mutant reads observed prior to INVAR analysis (when the bam file is summarized by pileup), in comparison to the number of reads observed after INVAR filtering.

INVAR (Integration of VAriant Reads, Wan et al, Science Translational Medicine, 2020) uses custom error-suppression and signal enrichment methods to enrich tumor-specific signal and suppress background noise, as described in Materials and Methods (lines 1085-1097). INVAR leverages custom error-suppression including (a) collapsing sequencing reads, (b) requiring every mutation to be both in a forward and a reverse read, (c) applying a locus noise filter and (d) applying a patient-specific outlier suppression that removes the signal from one locus if not consistent with the distribution of the remaining loci. In order to exclude possible germline SNPs, only loci with AFs < 0.25 are used (based on the assumption that if a large number of loci are tested in a high ctDNA sample the detection is supported by having many low AF loci with signal). Signal-enrichment methods included assigning greater weight to (e) loci with higher AF observed in the tumor and to (f) sequencing reads with a fragment-size similar to the size distribution of ctDNA in the analyzed cohort.

Table EV12 shows the number of reads (median, range) observed following SNV-hybrid capture, SNV-modWGS and SNV-deepWGS analysis of specific patient, unrelated patient and healthy control samples using patient-specific mutations lists before and after INVAR filtering. As shown, following INVAR analysis more mutant reads are observed in detected samples from the relevant patient than in undetected samples from the same patients, or in those from unrelated patients or in healthy control samples. Healthy and unrelated patient samples have a low number of mutant reads (median = 0 mutant reads), whereas detected samples showed a median of 808, 366 or 1002 mutant reads in SNV-hybrid capture, SNV-modWGS and SNV-deepWGS respectively across all samples analyzed.

For ctDNA assays targeting SVs:

SV-multiplex PCR

Each assay was tested on a pool of healthy donor cell-free DNA (cfDNA) with at least twice as many replicates as in test samples (Materials and Methods, lines 929-931). As indicated, we did observe a very low number of read counts (≤ 5) in negative controls, including healthy control cfDNA, and in samples from unrelated patients (Materials and Methods section, lines 1016– 1018), which may be due to index hopping. We present results of an experiment to test this hypothesis. Given SVs are gross genomic rearrangements, patient-specific somatic SVs are only expected to be identified in plasma DNA from the relevant patient, and not in unrelated patient or healthy control cfDNA. An explanation of how patient samples were classified as 'detected' or 'undetected' is indicated in Materials and Methods, lines 1010 – 1016.

To help further clarify, we have added the a sentence to the Results section, to point the reader to the Material and Methods section to find details of how data was analyzed and the detection criteria used (Results, lines 229-230):

SV-hybrid capture

In the Materials and Methods (lines 1055–1062), we highlight that low level signal (≤ 4 reads per SV) was observed in negative controls (including healthy control plasma), and in samples from unrelated patients, likely due to index hopping. We also include details of how patient samples were classified as 'detected' or 'undetected'.

To help further clarify, we have added the following (in bold) to the Results section, to point the reader to the Material and Methods section to find details of how data was analyzed and the detection criteria used (Results, lines 265-266):

SV-modWGS

In the Results section (lines 351-353) we highlight that no reads were observed in any healthy donor plasma sample, but were observed in 2 plasma samples from a different patient, which may be due to index-hopping of sample barcodes within the same sequencing lane.

SV-deepWGS

For this assay, each sample was sequenced in a different sequencing lane, which avoided index hopping as a source of noise. In Results (lines 361–362), we highlight that no non-specific reads were observed in any sample.

For ctDNA assays targeting SCNAs:

SCNA-sWGS, SCNA-modWGS and SCNA-deepWGS

In Materials and Methods (lines 1140-1141 and 1250-1257) and Results (lines 409), we highlight that data was analysed using ichorCNA using the author's recommended detection threshold of 3% tumor fraction. The same settings were used when analysing plasma from heatlhy donors and patients, tumor and buffy coat. Using this threshold, no SCNAs were detected in healthy control samples before size selection, and had lower ichorCNA values than the patient samples detected after size-selection.

Referee #3 (Comments on Novelty/Model System for Author):

The authors responded adequately to my remarks. It is an interesting work that deserves to be published even if it is not new and with a medical impact that can be qualified as medium.

We would like to thank Referree #3 for supporting publication of our manuscript.

2nd Mar 2023

Dear Dr. Gale,

Thank you for the submission of your revised manuscript to EMBO Molecular Medicine, which has been sent back to referee #2. As you will see below, this referee is overall supportive of publication pending addition of sequencing information pertaining to the structural variants.
I will therefore be able to accept your manuscript once you have addressed the remaining minor concerns from this referee.

Additionally, please provide a resized version of your synopsis picture (550px wide x 300-600 px high). I have tried to resize it myself, but the text gets blurry and difficult to read.

I look forward to receiving your revised manuscript.

With kind regards,

Lise Roth

Lise Roth, PhD
Senior Editor
EMBO Molecular Medicine

***** Reviewer's comments *****

Referee #2 (Comments on Novelty/Model System for Author):

As said before, the study is interesting because of the wide variety of technologies employed. However, the medical impact is still to be address including more patients and samples.

Referee #2 (Remarks for Author):

Many thanks to the authors for the revision version of their manuscript.
The authors have added a supplementary table 12 with partial information about sequencing stats obtained in samples and controls and clarify how they consider a sample as negative or positive for ctDNA detection. However, I still have concerns that must be addressed:

1.Regarding the sequencing stats for the SV assays, they refer in the text that are in Table EV12. In that table there is only information about SNVs assay. It is paramount to show sequencing information for these assays since is where they found one of the lowest AFs. In general, it is crucial to see the degree of noise in control samples in those SVs from positive samples where they observed the limit of detection.

2.The authors stated in lines 391-392 of the manuscript tracked version "The sample with the lowest AF detected was P-IV-03 plasma timepoint 6 with ctDNA detected at AF of 0.0016%." However, in Figure 3, that timepoint is indicated as "not detected".

3.Line 458-460 and 445-446: "All detected samples had a higher ichorCNA values than non-detected samples and healthy controls.". The values need to be shown.

Author's Point-by-Point response to Reviewers' comments

***** Reviewer's comments *****

Referee #2 (Comments on Novelty/Model System for Author):

As said before, the study is interesting because of the wide variety of technologies employed. However, the medical impact is still to be address including more patients and samples.

Referee #2 (Remarks for Author):

Many thanks to the authors for the revision version of their manuscript.
The authors have added a supplementary table 12 with partial information about sequencing stats obtained in samples and controls and clarify how they consider a sample as negative or positive for ctDNA detection. However, I still have concerns that must be addressed:

1.Regarding the sequencing stats for the SV assays, they refer in the text that are in Table EV12. In that table there is only information about SNVs assay. It is paramount to show sequencing information for these assays since is where they found one of the lowest AFs. In general, it is crucial

to see the degree of noise in control samples in those SVs from positive samples where they observed the limit of detection.

We would like to thank Referee #2 for general support our manuscript. We have now included Tables EV8 and Table EV11 to address Reviewer 2's concerns and show sequencing stats for the SV assays.

2.The authors stated in lines 391-392 of the manuscript tracked version "The sample with the lowest AF detected was P-IV-03 plasma timepoint 6 with ctDNA detected at AF of 0.0016%." However, in Figure 3, that timepoint is indicated as "not detected".

Please note that we highlighted the change in detection of Sample P-IV-03_T6 in our previous Rebuttal Letter (dated 5th March 2023), and corrected this sample to being undetected at >95% specificity. As a result, we updated appropriate Figures and Tables in the previous submission, and made appropriate changes to the manuscript. In the manuscript tracked version that we submitted (EMM-2022-16505-V3-Q_Manuscript_tracked), we see the sentence "The sample with the lowest AF detected was P-IV-03 plasma timepoint 6 with ctDNA detected at AF of 0.0016%." has been **deleted in Line 381-382**, as a result of this correction. Lines 391-392 are not relevant in this manuscript version.

3.Line 458-460 and 445-446: "All detected samples had a higher ichorCNA values than non-detected samples and healthy controls.". The values need to be shown.

Thank you for your comments. ichorCNA values for all patient plasma samples are shown in Table EV10 (was EV9), columns N-P. We have additionally summarised these in a new Table EV15 where we include the values for the healthy control plasma samples to address Reviewer 2's concerns and show ichorCNA values for samples and healthy controls using the the SCNA assays.

6th Apr 2023

Dear Dr. Gale,

Thank you for submitting your revised files. I am pleased to inform you that your manuscript is accepted for publication and is now being sent to our publisher to be included in the next available issue of EMBO Molecular Medicine!

We would like to remind you that as part of the EMBO Publications transparent editorial process initiative, EMBO Molecular Medicine will publish a Review Process File online to accompany accepted manuscripts. If you do NOT want the file to be published or would like to exclude figures, please immediately inform the editorial office via e-mail.

Please read below for additional IMPORTANT information regarding your article, its publication and the production process.

Congratulations on your interesting work,

With kind regards,

Lise Roth

Lise Roth, Ph.D
Senior Editor
EMBO Molecular Medicine

Follow us on Twitter @EmboMolMed
Sign up for eTOCs at embopress.org/alertsfeeds

*** *** *** IMPORTANT INFORMATION *** *** ***

SPEED OF PUBLICATION
The journal aims for rapid publication of papers, using using the advance online publication "Early View" to expedite the process: A properly copy-edited and formatted version will be published as "Early View" after the proofs have been corrected. Please help the Editors and publisher avoid delays by providing e-mail address(es), telephone and fax numbers at which author(s) can be contacted.

Should you be planning a Press Release on your article, please get in contact with embomolmed@wiley.com as early as possible, in order to coordinate publication and release dates.

LICENSE AND PAYMENT:

All articles published in EMBO Molecular Medicine are fully open access: immediately and freely available to read, download and share.

EMBO Molecular Medicine charges an article processing charge (APC) to cover the publication costs. You, as the corresponding author for this manuscript, should have already received a quote with the article processing fee separately. Please let us know in case this quote has not been received.

Once your article is at Wiley for editorial production you will receive an email from Wiley's Author Services system, which will ask you to log in and will present you with the publication license form for completion. Within the same system the publication fee can be paid by credit card, an invoice, pro forma invoice or purchase order can be requested.

Payment of the publication charge and the signed Open Access Agreement form must be received before the article can be published online.

PROOFS

You will receive the proofs by e-mail approximately 2 weeks after all relevant files have been sent o our Production Office. Please return them within 48 hours and if there should be any problems, please contact the production office at

embopressproduction@wiley.com.

Please inform us if there is likely to be any difficulty in reaching you at the above address at that time. Failure to meet our deadlines may result in a delay of publication.

All further communications concerning your paper proofs should quote reference number EMM-2022-16505-V5 and be directed to the production office at embopressproduction@wiley.com.

EMBO Press Author Checklist

Corresponding Author Name: Dr Davina Gale	
Journal Submitted to: EMBO Molecular Medicine	
Manuscript Number: EMM-2022-16505-V2-Q	

USEFUL LINKS FOR COMPLETING THIS FORM
The EMBO Journal - Author Guidelines
EMBO Reports - Author Guidelines
Molecular Systems Biology - Author Guidelines
EMBO Molecular Medicine - Author Guidelines

Reporting Checklist for Life Science Articles (updated January
This checklist is adapted from Materials Design Analysis Reporting (MDAR) Checklist for Authors. MDAR establishes a minimum set of requirements in transparent reporting in the life sciences (see Statement of Task: 10.31222/osf.io/9sm4x). Please follow the journal's guidelines in preparing your
Please note that a copy of this checklist will be published alongside your article.

Abridged guidelines for figures
1. Data
The data shown in figures should satisfy the following conditions:
- the data were obtained and processed according to the field's best practice and are presented to reflect the results of the experiments in an accurate and unbiased manner.
- ideally, figure panels should include only measurements that are directly comparable to each other and obtained with the same assay.
- plots include clearly labeled error bars for independent experiments and sample sizes. Unless justified, error bars should not be shown for technical
- if n<5, the individual data points from each experiment should be plotted. Any statistical test employed should be justified.
- Source Data should be included to report the data underlying figures according to the guidelines set out in the authorship guidelines on Data

2. Captions
Each figure caption should contain the following information, for each panel where they are relevant:
- a specification of the experimental system investigated (eg cell line, species name).
- the assay(s) and method(s) used to carry out the reported observations and measurements.
- an explicit mention of the biological and chemical entity(ies) that are being measured.
- an explicit mention of the biological and chemical entity(ies) that are altered/varied/perturbed in a controlled manner.
- the exact sample size (n) for each experimental group/condition, given as a number, not a range;
- a description of the sample collection allowing the reader to understand whether the samples represent technical or biological replicates (including how many animals, litters, cultures, etc.).
- a statement of how many times the experiment shown was independently replicated in the laboratory.
- definitions of statistical methods and measures:
  - common tests, such as t-test (please specify whether paired vs. unpaired), simple χ2 tests, Wilcoxon and Mann-Whitney tests, can be unambiguously identified by name only, but more complex techniques should be described in the methods section;
  - are tests one-sided or two-sided?
  - are there adjustments for multiple comparisons?
  - exact statistical test results, e.g., P values = x but not P values < x;
  - definition of 'center values' as median or average;
  - definition of error bars as s.d. or s.e.m.

Please complete ALL of the questions below.
Select "Not Applicable" only when the requested information is not relevant for your study.

Materials

Newly Created Materials	Information included in the manuscript?	In which section is the information available? (Reagents and Tools Table, Materials and Methods, Figures, Data Availability Section)
New materials and reagents need to be available; do any restrictions apply?	Not Applicable	

Antibodies	Information included in the manuscript?	In which section is the information available? (Reagents and Tools Table, Materials and Methods, Figures, Data Availability Section)
For **antibodies** provide the following information:<br>- Commercial antibodies: RRID (if possible) or supplier name, catalogue number and or/clone number<br>- Non-commercial: RRID or citation	Not Applicable	

DNA and RNA sequences	Information included in the manuscript?	In which section is the information available? (Reagents and Tools Table, Materials and Methods, Figures, Data Availability Section)
**Short novel DNA or RNA including primers, probes**: provide the sequences.	Yes	Table EV5. Patient-specific primers used in the SV-multiplex PCR assay.

Cell materials	Information included in the manuscript?	In which section is the information available? (Reagents and Tools Table, Materials and Methods, Figures, Data Availability Section)
**Cell lines:** Provide species information, strain. Provide accession number in repository **OR** supplier name, catalog number, clone number, and/**OR** RRID.	Not Applicable	
**Primary cultures:** Provide species, strain, sex of origin, genetic modification status.	Not Applicable	
Report if the cell lines were recently **authenticated** (e.g., by STR profiling) and tested for mycoplasma contamination.	Not Applicable	

Experimental animals	Information included in the manuscript?	In which section is the information available? (Reagents and Tools Table, Materials and Methods, Figures, Data Availability Section)
**Laboratory animals or Model organisms:** Provide species, strain, sex, age, genetic modification status. Provide accession number in repository **OR** supplier name, catalog number, clone number, **OR** RRID.	Not Applicable	
**Animal observed in or captured from the field:** Provide species, sex, and age where possible.	Not Applicable	
Please detail **housing and husbandry conditions**.	Not Applicable	

Plants and microbes	Information included in the manuscript?	In which section is the information available? (Reagents and Tools Table, Materials and Methods, Figures, Data Availability Section)
**Plants:** provide species and strain, ecotype and cultivar where relevant, unique accession number if available, and source (including location for collected wild specimens).	Not Applicable	
**Microbes:** provide species and strain, unique accession number if available, and source.	Not Applicable	

Human research participants	Information included in the manuscript?	In which section is the information available? (Reagents and Tools Table, Materials and Methods, Figures, Data Availability Section)
If collected and within the bounds of privacy constraints report on age, sex and gender or ethnicity for all study participants.	Yes	Table EV1. Clinical information on the breast cancer patients and samples analysed

Core facilities	Information included in the manuscript?	In which section is the information available? (Reagents and Tools Table, Materials and Methods, Figures, Data Availability Section)
If your work benefited from core facilities, was their service mentioned in the acknowledgments section?	Yes	Acknowledgments

Design

Study protocol	Information included in the manuscript?	In which section is the information available? (Reagents and Tools Table, Materials and Methods, Figures, Data Availability Section)

If study protocol has been **pre-registered, provide DOI in the manuscript**. For clinical trials, provide the trial registration number **OR** cite DOI.	Yes	Materials and Methods: Ethics approval and consent to participate
Report the **clinical trial registration number** (at ClinicalTrials.gov or equivalent), where applicable.	Not Applicable	

Laboratory protocol	Information included in the manuscript?	In which section is the information available? (Reagents and Tools Table, Materials and Methods, Figures, Data Availability Section)
Provide DOI OR other citation details if **external detailed step-by-step protocols** are available.	Not Applicable	

Experimental study design and statistics	Information included in the manuscript?	In which section is the information available? (Reagents and Tools Table, Materials and Methods, Figures, Data Availability Section)
Include a statement about **sample size** estimate even if no statistical methods were used.	Yes	Materials and Methods : Statistical considerations
Were any steps taken to minimize the effects of subjective bias when allocating animals/samples to treatment (e.g. **randomization procedure**)? If yes, have they been described?	Yes	Materials and Methods : Statistical considerations
Include a statement about **blinding** even if no blinding was done.	Yes	Materials and Methods : Statistical considerations
Describe **inclusion/exclusion criteria** if samples or animals were excluded from the analysis. Were the criteria pre-established?<br><br>If sample or data points were omitted from analysis, report if this was due to attrition or intentional exclusion and provide justification.	Yes	Materials and Methods : Statistical considerations
For every figure, are **statistical tests** justified as appropriate? Do the data meet the assumptions of the tests (e.g., normal distribution)? Describe any methods used to assess it. Is there an estimate of variation within each group of data? Is the variance similar between the groups that are being statistically compared?	Yes	Materials and Methods : Statistical considerations

Sample definition and in-laboratory replication	Information included in the manuscript?	In which section is the information available? (Reagents and Tools Table, Materials and Methods, Figures, Data Availability Section)
In the figure legends: state number of times the experiment was **replicated** in laboratory.	Not Applicable	
In the figure legends: define whether data describe **technical or biological replicates**.	Not Applicable	

Ethics

Ethics	Information included in the manuscript?	In which section is the information available? (Reagents and Tools Table, Materials and Methods, Figures, Data Availability Section)
Studies involving **human participants**: State details of **authority granting ethics approval** (IRB or equivalent committee(s), provide reference number for approval.	Yes	Materials and Methods : Ethics approval and consent to participate
Studies involving **human participants**: Include a statement confirming that **informed consent** was obtained from all subjects and that the experiments conformed to the principles set out in the WMA Declaration of Helsinki and the Department of Health and Human Services Belmont Report.	Yes	Materials and Methods : Ethics approval and consent to participate
Studies involving **human participants:** For publication of **patient photos**, include a statement confirming that consent to publish was obtained.	Not Applicable	
Studies involving experimental **animals**: State details of **authority granting ethics approval** (IRB or equivalent committee(s), provide reference number for approval. Include a statement of compliance with ethical regulations.	Not Applicable	
Studies involving **specimen and field samples:** State if relevant **permits** obtained, provide details of authority approving study; if none were required, explain why.	Not Applicable	

Dual Use Research of Concern (DURC)	Information included in the manuscript?	In which section is the information available? (Reagents and Tools Table, Materials and Methods, Figures, Data Availability Section)
Could your study fall under dual use research restrictions? Please check biosecurity documents and list of **select agents and toxins** (CDC): https://www.selectagents.gov/sat/list.htm	Not Applicable	
If you used a select agent, is the security level of the lab appropriate and reported in the manuscript?	Not Applicable	
If a study is subject to dual use research of concern regulations, is the name of the **authority granting approval and reference number** for the regulatory approval provided in the manuscript?	Not Applicable	

Reporting

The MDAR framework recommends adoption of discipline-specific guidelines, established and endorsed through community initiatives. Journals have their own policy about requiring specific guidelines and recommendations to complement MDAR.

Adherence to community standards	Information included in the manuscript?	In which section is the information available? (Reagents and Tools Table, Materials and Methods, Figures, Data Availability Section)
State if relevant guidelines or checklists (e.g., **ICMJE, MIBBI, ARRIVE, PRISMA**) have been followed or provided.	Not Applicable	
For **tumor marker prognostic studies,** we recommend that you follow the **REMARK** reporting guidelines (see link list at top right). See author guidelines, under 'Reporting Guidelines'. Please confirm you have followed these guidelines.	Not Applicable	
For **phase II and III randomized controlled trials**, please refer to the **CONSORT** flow diagram (see link list at top right) and submit the CONSORT checklist (see link list at top right) with your submission. See author guidelines, under 'Reporting Guidelines'. Please confirm you have submitted this list.	Not Applicable	

Data Availability

Data availability	Information included in the manuscript?	In which section is the information available? (Reagents and Tools Table, Materials and Methods, Figures, Data Availability Section)
Have **primary datasets** been deposited according to the journal's guidelines (see 'Data Deposition' section) and the respective accession numbers provided in the Data Availability Section?	Yes	Data availability
Were **human clinical and genomic datasets** deposited in a public access-controlled repository in accordance to ethical obligations to the patients and to the applicable consent agreement?	Yes	Data availability
Are **computational models** that are central and integral to a study available without restrictions in a machine-readable form? Were the relevant accession numbers or links provided?	Yes	Data availability
If publicly available data were reused, provide the respective **data citations in the reference list**.	Not Applicable	
